# Association of Visual Health With Depressive Symptoms and Brain Imaging Phenotypes Among Middle-Aged and Older Adults

**DOI:** 10.1001/jamanetworkopen.2022.35017

**Published:** 2022-10-06

**Authors:** Xiayin Zhang, Xianwen Shang, Ishith Seth, Yu Huang, Yaxin Wang, Yingying Liang, Zijing Du, Guanrong Wu, Yunyan Hu, Shunming Liu, Yijun Hu, Mingguang He, Zhuoting Zhu, Xiaohong Yang, Honghua Yu

**Affiliations:** 1 Guangdong Eye Institute, Department of Ophthalmology, Guangdong Provincial People’s Hospital, Guangdong Academy of Medical Sciences, Guangzhou, China; 2 Guangdong Cardiovascular Institute, Guangdong Provincial People’s Hospital, Guangdong Academy of Medical Sciences, Guangzhou, China; 3 Centre for Eye Research Australia, Royal Victorian Eye and Ear Hospital, East Melbourne, Victoria, Australia; 4 State Key Laboratory of Ophthalmology, Zhongshan Ophthalmic Center, Sun Yat-sen University, Guangzhou, China

## Abstract

**Importance:**

Vision loss and depression are common conditions with major health implications. However, mechanisms of the association of visual health (across the full acuity spectrum) with depression remain unclear.

**Objective:**

To characterize the association between visual health and depression and investigate the association between depression and brain microstructure and macrostructure in subgroups divided by visual acuity.

**Design, Setting, and Participants:**

In the UK Biobank Study cohort, 114 583 volunteers were included at baseline from March to June 2006 to July 2010. Habitual distance visual acuity was examined using the logarithm of the minimum angle of resolution (LogMAR) characters. Depression was identified based on Patient Health Questionnaire (PHQ) or through an interview-based psychiatric diagnosis. Subgroup participants completed multimodal magnetic resonance imaging (MRI) of the brain and PHQ evaluation during the imaging visit after 2014. Data were analyzed from May 5 to August 9, 2022.

**Main Outcomes and Measures:**

Depression, depressive symptoms, and imaging-derived phenotypes from T1-weighted and diffusion MRI.

**Results:**

Of the 114 583 participants from the UK Biobank Study, 62 401 (54.5%) were women, and the mean (SD) age was 56.8 (8.1) years (range, 39-72 years). A 1-line worse visual acuity (0.1 LogMAR increase) was associated with 5% higher odds of depression (odds ratio, 1.05 [95% CI, 1.04-1.07]) after adjustment for age, sex, race and ethnicity, Townsend index, educational qualifications, smoking, alcohol consumption, obesity, physical activity, history of hypertension, diabetes, hyperlipidemia, and family history of depression. Of the 7844 participants eligible for MRI analysis, there were linear associations between PHQ score and the left volume of gray matter in supracalcarine cortex (coefficient, 7.61 [95% CI, 3.90-11.31]) and mean isotropic volume fraction (ISOVF) in the right fornix (cres) and/or stria terminalis (coefficient, 0.003 [95% CI, 0.001-0.004]) after correction for multiple comparison. In addition, their association could be moderated by visual acuity, whereby increased PHQ score was associated with higher ISOVF levels only among those with poorer visual acuity (*P* = .02 for interaction).

**Conclusions and Relevance:**

This study suggests an association between visual health and depression and that the diffusion characteristic of ISOVF in the fornix (cres) and/or stria terminalis is associated with depressive symptoms in participants with poorer visual acuity.

## Key Points

**Question:**

Is visual health across the full acuity spectrum associated with depressive symptoms and corresponding changes in brain imaging signatures among middle-aged and older adults?

**Findings:**

In this cohort study of 114 583 participants from the UK Biobank Study, poorer visual acuity was associated with depressive symptoms measured by Patient Health Questionnaire score across the full spectrum. In addition, poorer visual acuity exacerbated the linear associations between Patient Health Questionnaire score and extracellular water diffusion in the fornix (cres) and/or stria terminalis.

**Meaning:**

These findings suggest that visual health is associated with depressive symptoms and altered brain neurobiology and that health care practitioners should consider these outcomes when performing routine mental health screening.

## Teaser

This cohort study characterizes alterations in brain structures due to depressive symptoms and assesses their association with poorer visual health in middle-aged and older adults from the UK Biobank Study.

## Introduction

Good visual functioning is essential to satisfaction and value among older adults. With advanced aging, vision-related problems have major public health implications^1^ and contribute to difficulties in activities of daily living, morbidity, and mortality in middle-aged and older adults.^2,3,4,5^ It is well known that visual impairment contributes to the burden of depression.^6,7^ The mechanisms underlying their relationship are complex, but 2 main pathways can be identified. First, poor eye conditions and depression share common risk factors, including poverty.^8,9^ Second, visual impairment may cause depression owing to difficulties in reading, mobility, and driving.^10,11,12,13^ A previous meta-analysis^14^ indicated that 1 in 4 elderly patients with visual impairment reported depression. However, the association between depression and the full spectrum of visual acuity remains unclear.

Depression is another major burden to society that is projected to rank as the primary cause of burden of disease worldwide by 2030.^15^ Depression in older adults is subtle and often underrecognized and undertreated, whereas the current pharmacological treatment is generally effective against severe depression.^16,17^ Therefore, the management of poorer visual acuity may help to reduce the prevalence of depression. It has been shown that depression also causes alterations in brain structures and functional connections.^18^ Conventional magnetic resonance imaging (MRI) has been used to investigate pathological changes, and diffusion MRI (dMRI) allows mapping of cortical connections.^19^ Meta-analyses using MRI and dMRI^20,21^ have demonstrated that depression is associated with volumetric reductions in the hippocampus, lower fractional anisotropy, and higher radial diffusivity. Because poorer visual acuity and depression can coexist in individuals, further research is needed to understand the brain changes and neural mechanisms linked between them.

The present study aims to investigate the association between visual health (across the visual acuity spectrum) and depressive symptoms in a large sample of middle-aged and older adults from the UK Biobank cohort. The combination of a large participant cohort and multimodal imaging data is a unique feature of the UK Biobank Study,^22^ which offers information on imaging-derived phenotypes (IDPs) of brain macrostructure and microstructure and additionally estimates the neurite density (ie, intracellular volume fraction), extracellular water diffusion (ie, isotropic volume fraction [ISOVF]), and tract complexity and/or fanning (ie, orientation dispersion).^23,24^ We aimed to characterize alterations in brain structures due to depressive symptoms measured by the Patient Health Questionnaire (PHQ) tool. We hypothesized that poorer visual acuity would be associated with depression and may alter the depression-related brain structures.

## Methods

### Data

From March to June 2006 to July 2010, the UK Biobank Study recruited 502 205 participants aged 39 to 73 years. At baseline, all participants completed standardized questionnaires and ocular examinations. The UK Biobank Study’s ethical approval was granted by the North West Multi-center Research Ethics Committee. Our access to data from the UK Biobank cohort was approved by the UK Biobank Ethics Advisory Committee. All participants provided informed consent. The study was performed according to the principles of the Declaration of Helsinki and followed the Strengthening the Reporting of Observational Studies in Epidemiology (STROBE) reporting guideline.

### Measurement of Visual Acuity

The ocular examinations included distance visual acuity measured in the logarithm of the minimum angle of resolution (LogMAR) using commercially available charts and devices.^25^ The distance visual acuity was conducted with participants wearing any prescribed optical correction as required at 1 or 4 m. Participants were asked to read each letter from the end of each line from top to bottom, and the evaluation was terminated when 2 or more letters were incorrect. Visual impairment was defined as visual acuity worse than 0.3 LogMAR units.

### Evaluation of Depression

At baseline between 2006 and 2010, depressive symptoms were assessed through self-reported symptoms (2-item PHQ [PHQ-2]).^26^ An interview-based assessment of depression diagnosis by a medical practitioner was also completed at baseline. Thus, depression was determined by a positive answer to the interview-based question or a PHQ score of 3 or more.^27,28^ The participants also completed PHQ-2 tests during the neuroimaging visit after 2014.^29^

During an online follow-up in 2016, the 9-item PHQ (PHQ-9) was sent to the UK Biobank participants (n = 339 092), and 157 367 responded (response rate, 46.4%). The PHQ-9 consists of 9 criteria of depressive symptoms with scores ranging from 0 to 27, with scores above 10 indicating depression.^30,31^ A direct comparison of the diagnostic ability of PHQ-2 and PHQ-9 showed that they perform similarly with sensitivities of 98% and specificities of 80% in the screening of major depressive disorder.^32^

### MRI Acquisition and IDP Processing

Brain MRI was acquired on a standard 3T scanner (Skyra; Siemens) (eMethods in the Supplement). Total brain volume white matter and gray matter volumes (WMV and GMV) and regional GMV were acquired from T1-weighted structural MRI. The microstructure of white matter showing cortical connection was quantified by dMRI. In addition to conventional measures of the directional coherence (fractional anisotropy) and the magnitude of water molecule diffusion, the newer neurite orientation dispersion and density imaging^33^ that measures white matter microstructure of neurite density (intracellular volume fraction), extracellular water diffusion (ISOVF), and tract complexity and/or fanning (orientation dispersion) were also obtained.^34^ The resultant parameters were made available as IDPs (eTable 1 in the Supplement).

**Figure 1.**
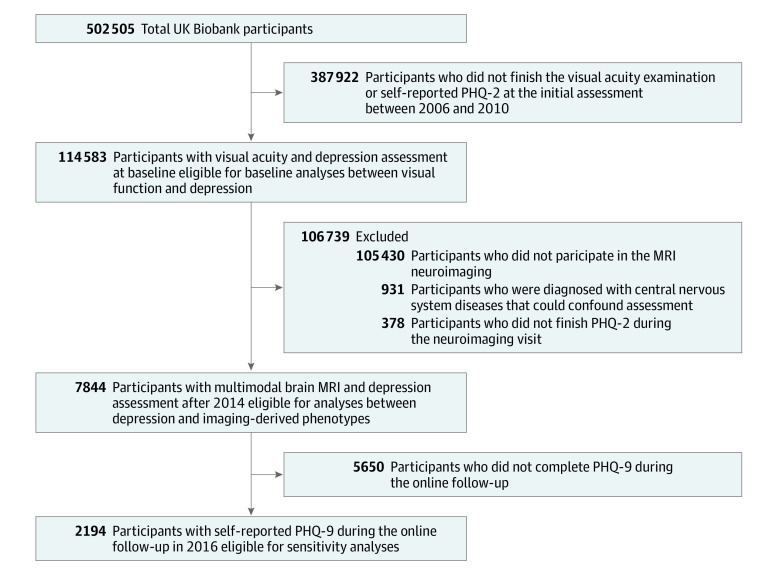
Selection of Eligible Participants From the UK Biobank Cohort Abbreviations: MRI, magnetic resonance imaging; PHQ-2, 2-item Patient Health Questionnaire; PHQ-9, 9-item PHQ.

Participants with images badly affected by movement artifacts were removed. In addition, participants with self-reported diagnosis of neurological diseases, including stroke, dementia, Parkinson disease, or any other demyelinating or neurodegenerative disorder, were excluded. The flowchart in Figure 1 illustrates the inclusion of participants.

### Demographic Data

Demographic information included age, sex, and Townsend index. Race and ethnicity data were self-reported and recorded as Asian, Black, Chinese, White, and multiple races or ethnicities. Race and ethnicity data were collected because prevalences of visual loss and depression vary among racial and ethnic groups. Other covariates, including educational qualifications, smoking, alcohol consumption, physical activity, and family history of severe depression, were obtained through standardized questionnaires. Obesity was defined as body mass index of greater than 30 (calculated as weight in kilograms divided by height in meters squared). Diabetes, hypertension, and hyperlipidemia were defined by self-report, diagnoses, medications, or physical measurements (eMethods in the Supplement).

### Statistical Analysis

Data were analyzed from May 5 to August 9, 2022. Continuous variables normally distributed were reported using mean (SD) or median (IQR), whereas categorical variables were summarized as count (percentage). Age- and sex-adjusted logistic regression models were applied to identify baseline characteristics associated with visual impairment or depression.

Logistic regression models were applied to evaluate the association between depression and visual acuity or visual impairment at baseline. Negative binomial regression models were applied to evaluate the association between PHQ-2 scores and visual acuity or visual impairment, owing to the positive skew in the distribution of PHQ-2 scores (eFigure 1A in the Supplement). For each analysis, age, sex, race and ethnicity, Townsend index, educational qualifications, smoking, alcohol consumption, obesity, physical activity, history of hypertension, diabetes, hyperlipidemia, and family history of depression were used as covariates. In addition, restricted cubic spline analyses of nonlinear associations between visual acuity and depressive symptoms were performed, with 4 knots placed at equal percentiles of visual acuity.

We further explored the association between depressive symptoms and brain structures using linear regression. Covariate adjustments were conducted in stages, starting with a basic model (age, sex, race and ethnicity, and obesity) and then fully adjusted for all covariates. Adjusted *P* values were calculated using false discovery rate methods. Nonlinear relationships were estimated using restricted cubic spline models. Visual acuity was derived into 4 category variables to assess the role of visual acuity on the association between depression and brain structure. We performed all analyses for the total sample stratified by age and sex. Finally, the analysis was repeated using PHQ-9 in sensitivity analyses. In addition, Spearman correlation analysis was performed to investigate the association between PHQ-2 and PHQ-9 scores.

All statistical analyses were conducted using Stata, version 16.0 (StataCorp LLC), and R, version 3.3.0 (R Foundation for Statistical Computing). Two-sided *P* < .05 indicated statistical significance.

## Results

### Included Participants

A total of 502 505 people were recruited from 22 centers into the UK Biobank cohort study at baseline. Our baseline analyses included 114 583 participants with a mean (SD) age of 56.8 (8.1) years (range, 39-72 years), with 62 401 women (54.5%) and 52 182 men (45.5%) completing both visual screening and mental tasks (Figure 1).

Of the 114 583 participants, 99 871 (87.2%) had no visual impairment or depression (healthy controls), 3667 (3.2%) showed visual impairment, 11 500 (10.0%) reported depression diagnosis, and 455 (0.4%) had both. Participant characteristics are summarized in Table 1. Participants with visual impairment were more likely to be older (mean [SD] age, 58.8 [7.5] vs 56.7 [8.1] years), to be from a racial or ethnic minority group (614 [16.7%] vs 11 659 [10.5%]), to have lower socioeconomic (mean [SD] Townsend index, −0.18 [3.32] vs −0.98 [2.99]) and educational status (2702 [73.7%] vs 72 038 [64.9%]), to use alcohol less (3336 [91.0%] vs 105 001 [94.8%]), and to not have hyperlipidemia (2098 [57.2%] vs 64 785 [58.4%]). However, participants with visual impairment had higher rates of obesity (963 [26.3%] vs 26 958 [24.3%]), diabetes (329 [9.0%] vs 7228 [6.5%]), and hypertension (2898 [79.0%] vs 82 269 [74.2%]). In the analysis stratified by depression, depression was also associated with a range of health and lifestyle covariates (eTable 2 in the Supplement).

**Table 1.** Baseline Characteristics of Study Participants

Characteristic	Participant group^a^			OR (95% CI)^b^
	Total (N = 114 583)	No visual impairment (n = 110 916)	Visual impairment (n = 3667)	
Age, mean (SD), y	56.8 (8.1)	56.7 (8.1)	58.8 (7.5)	1.03 (1.03-1.04)^c^
Sex				
Women	62 401 (54.5)	60 366 (54.4)	2035 (55.5)	1 [Reference]
Men	52 182 (45.5)	50 550 (45.6)	1632 (44.5)	0.94 (0.88-1.00)
Race and ethnicity				
Non-White^d^	12 273 (10.7)	11 659 (10.5)	614 (16.7)	2.00 (1.83-2.19)^c^
White	102 310 (89.3)	99 257 (89.5)	3053 (83.3)	1 [Reference]
Townsend index, mean (SD)	−0.95 (3.00)	−0.98 (2.99)	−0.18 (3.32)	1.10 (1.09-1.11)^c^
Educational level				
College or university degree	39 843 (34.8)	38 878 (35.1)	965 (26.3)	1 [Reference]
Other	74 740 (65.2)	72 038 (65.0)	2702 (73.7)	1.42 (1.32-1.53)^c^
Smoking status				
Never	63 332 (55.5)	61 323 (55.5)	2009 (55.3)	1 [Reference]
Former or current	50 736 (44.5)	49 115 (44.5)	1621 (44.7)	0.97 (0.90-1.03)
Use of alcohol				
Never	6023 (5.3)	5716 (5.2)	307 (8.4)	1 [Reference]
Former or current	108 337 (94.7)	105 001 (94.8)	3336 (91.0)	0.58 (0.52-0.66)^c^
Obesity				
No	86 018 (75.5)	83 372 (75.6)	2646 (73.3)	1 [Reference]
Yes	27 921 (24.5)	26 958 (24.4)	963 (26.7)	1.12 (1.04-1.20)^c^
Physical activity				
Not meeting recommendation	16 514 (17.7)	15 988 (17.7)	526 (18.3)	1 [Reference]
Meeting recommendation	76 790 (82.3)	74 443 (82.3)	2347 (81.7)	0.93 (0.85-1.02)
Family history of depression				
No	99 300 (86.7)	96 103 (86.6)	3197 (87.2)	1 [Reference]
Yes	15 283 (13.3)	14 813 (13.3)	470 (12.8)	0.97 (0.88-1.07)
History of diabetes				
No	107 026 (93.4)	103 688 (93.5)	3338 (91.0)	1 [Reference]
Yes	7557 (6.6)	7228 (6.5)	329 (9.0)	1.32 (1.17-1.48)^c^
History of hypertension				
No	29 416 (25.7)	28 647 (25.8)	769 (21.0)	1 [Reference]
Yes	85 167 (74.3)	82 269 (74.2)	2898 (79.0)	1.15 (1.06-1.25)^c^
History of hyperlipidemia				
No	66 883 (58.4)	64 785 (58.4)	2098 (57.2)	1 [Reference]
Yes	47 700 (41.6)	46 131 (41.6)	1569 (42.8)	0.93 (0.87-0.99)^c^

Abbreviations: LogMAR, logarithm of the minumum angle of resolution; OR, odds ratio.

^a^ Unless indicated otherwise, data are expressed as No. (%) of participants. Percentages have been rounded and therefore may not total 100. Data were incomplete for some characteristics and therefore total less than numbers in the column headings. No visual impairment is defined as presenting visual acuity 0.3 LogMAR or less in the better-seeing eye; visual impairment is defined as presenting visual acuity greater than 0.3 LogMAR in the better-seeing eye.

^b^ Logistic regression models are adjusted for age and sex.

^c^ *P* < .05.

^d^ Includes Asian, Black, Chinese, and multiple races or ethnicities.

A subset of 8775 individuals who underwent assessment with PHQ-2 and brain MRI between 2014 and 2019 were included to explore the association between depressive symptoms and brain structures. Participants were excluded if they had been diagnosed with neurological pathology (n = 931) Figure 1). Of the 7844 participants included in this analysis, 4004 (51.0%) were women, 3840 (49.0%) were men, and 153 (2.0%) had visual impairment. The distributions of total brain volume, GMV, and WMV are provided in eTable 3 in the Supplement.

### Associations Between Visual Health and Depression

Among the 3667 adults with visual impairment, 455 (12.4%) had depression compared with 10 981 of 110 916 (9.9%) without visual impairment. The associations between visual health and depression are presented in Table 2. After adjusting for potential confounders, visual impairment was associated with a 19% higher risk of depression (odds ratio, 1.19; [95% CI, 1.05-1.34]; *P* = .003). A 1-line worse visual acuity (0.1-LogMAR increase) was associated with 5% higher odds of depression (odds ratio, 1.05 [95% CI, 1.04-1.07]; *P* < .001). Specifically, a 1-line worse visual acuity was also associated with greater PHQ-2 scores (adjusted incidence rate ratio, 1.04 [95% CI, 1.03-1.05]; *P* < .001). Restricted cubic spline analysis does not consider nonlinear associations between visual acuity and PHQ-2 scores (nonlinear *P* = .07) (eFigure 1B in the Supplement).

**Table 2.** Results of Covariate-Adjusted Regression Analyses Showing Associations Between Visual Health and Depression^a^

Outcome	Instrument	OR (95% CI)^b^	IRR (95% CI)^c^	*P* value
Depression^d^	Visual acuity (continuous variable, per 0.1 LogMAR units)	1.05 (1.04-1.07)	NA	<.001
	Visual impairment (categorical variable)	1.19 (1.05-1.34)	NA	.003
Depressive symptoms (PHQ-2)	Visual acuity (continuous variable, per 0.1 LogMAR units)	NA	1.04 (1.03-1.05)	<.001
	Visual impairment (categorical variable)	NA	1.15 (1.07-1.23)	<.001

Abbreviations: IRR, incident rate ratio; LogMAR, logarithm of the minimum angle of resolution; NA, not applicable; OR, odds ratio; PHQ-2, 2-item Patient Health Questionnaire.

^a^ All models were adjusted for age, sex, race and ethnicity, Townsend index, educational qualifications, smoking, alcohol consumption, obesity, physical activity, history of hypertension, diabetes, hyperlipidemia, and family history of depression.

^b^ Logistic regression models were used to test the association between depression status (categorical variable) and visual impairment.

^c^ Negative binomial regression models were used to test the association between PHQ-2 score (continuous variable) and visual impairment.

^d^ Determined by a positive answer to the self-reported question or a score of 3 or more on the PHQ-2 tool.

Further analysis of depression was stratified by age (median, 58 [IQR, 50-63] years) into younger (39-58 years) and older (59-72 years) cohorts. The association between visual acuity and depression observed in the entire cohort remained present in both groups (eTable 4 in the Supplement). In analyses stratified by sex, the patterns of association between visual acuity and depression were also consistent with those of the whole population (eTable 4 in the Supplement).

### Association of Depressive Symptoms With Brain Macrostructure and Microstructure

We conducted analyses across brain macrostructure and microstructure, including 3 global IDPs (total cerebral volume, GMV, and WMV), 139 regional GMV IDPs, and 375 white matter microstructure IDPs. We normalized all IDPs for head size by multiplying the raw IDP by the head size scaling factor.

**Figure 2.**
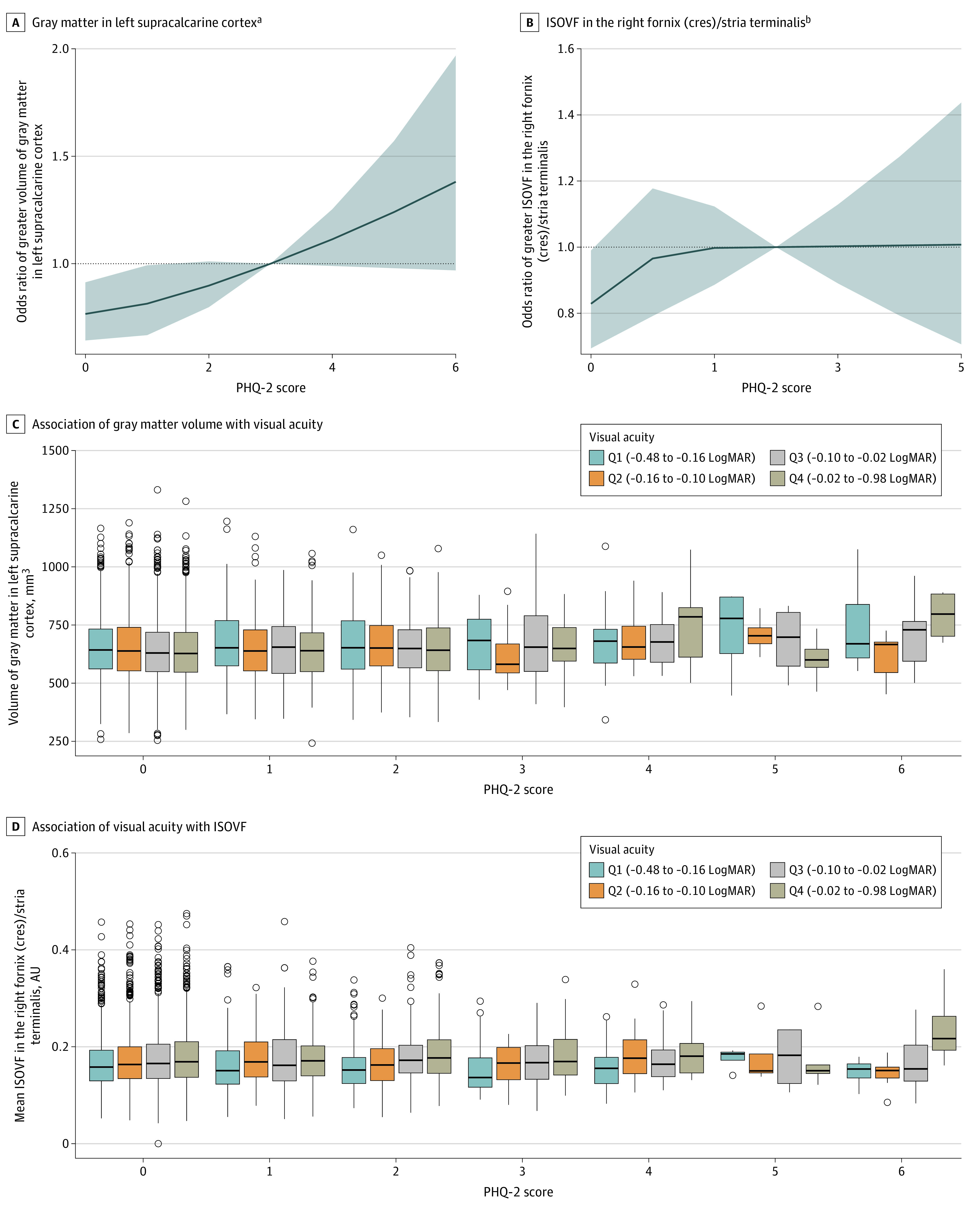
Association Between 2-Item Patient Health Questionnaire (PHQ-2) Score and Magnetic Resonance Imaging (MRI)–Derived Indices of Interest A and B, Restricted cubic spline curves show adjusted odds ratios and 95% CIs (gray shading) for greater gray matter volume (GMV) in the left supracalcarine cortex (A) and greater isotropic volume fraction (ISOVF) in the right fornix (cres) and/or stria terminalis (B) associated with PHQ-2 score. C, For GMV in the left supracalcarine cortex, among those in the highest and lowest quartile of visual acuity, a greater PHQ-2 score was associated with higher volume. D, For mean ISOVF in the right fornix (cres) and/or stria terminalis, greater PHQ-2 scores were associated with higher ISOVF levels in those with poorer visual acuity. LogMAR indicates logarithm of the minimum angle of resolution. ^a^*P* for linear trend < .001; *P* for nonlinear trend = .20. ^b^*P* for linear trend < .001; *P* for nonlinear trend = .23.

In primary analyses that controlled for age, sex, race and ethnicity, and obesity, we observed no associations with total cerebral volume, global GMV, or WMV. Associations between regional GMV IDPs and PHQ-2 scores are provided in eTable 5 in the Supplement, and associated patterns between white matter microstructure IDPs and PHQ-2 scores are provided in eFigure 2 in the Supplement. In secondary analyses that controlled for all covariates, the GMV in the left supracalcarine cortex was still associated with PHQ-2 scores (coefficient, 7.61 [95% CI, 3.90-11.31]; adjusted *P* = .006). In addition, mean ISOVF in the right fornix (cres) and/or stria terminalis showed an association with PHQ-2 scores after adjustment (coefficient, 0.003 [95% CI, 0.001-0.004]; adjusted *P* = .01). The linear associations between PHQ-2 scores with supracalcarine cortex volume and extracellular water diffusion in the fornix (cres)/stria terminalis are shown in Figure 2A and B, indicating deterioration in brain structures with greater depressive symptoms.

In age-stratified analysis, the associations did not differ by age for associations between depression, supracalcarine cortex volume, and extracellular water diffusion in the fornix (cres) and/or stria terminalis (coefficients, −7.39 and 0.01; *P* = .58 and *P* = .47 for interaction, respectively). In sex-stratified analysis, the association between the PHQ-2 score and the GMV in the supracalcarine cortex was only present among women (*P* = .03 for interaction). In contrast, depressive symptoms were associated with ISOVF in the fornix (cres) and/or stria terminalis only among men (*P* = .04 for interaction) (eTable 6 in the Supplement).

### Vision Modification of Depressive Symptoms and Brain Structure Associations

We next assessed whether the association between depressive symptoms and the 2 associated IDPs could be modified by visual acuity. We found evidence of modification of visual acuity as a continuous variable on the association between PHQ-2 score and ISOVF in the fornix (cres) and/or stria terminalis (*P* = .04 for interaction), but not on the association with GMV in supracalcarine cortex (*P* = .64 for interaction).

To visualize the associations between the 2 associated IDPs and the PHQ-2 score in subsamples binned according to the visual acuity range, Figure 2D and Table 3 illustrate that among those with poorer visual acuity (highest 2 quartiles), increased depressive symptoms were associated with higher ISOVF levels (coefficients, 5.8 × 10^−3^ and 4.6 × 10^−3^; *P* = .001 and *P* = .002, respectively). However, among those with better visual acuity (lowest 2 quartiles), depressive symptoms were not associated with ISOVF (coefficients, −3.5 × 10^−5^ and 1.7 × 10^−3^; *P* = .98 and, *P* = .25, respectively). For the GMV in the left supracalcarine cortex, only in the highest and lowest quartile of visual acuity, a greater PHQ-2 was associated with higher volume (coefficients, 12.80 and 10.38; *P* = .001 and *P* = .006, respectively) (Figure 2C).

**Table 3.** Covariate-Adjusted Linear Regression Analyses for the Associations Between PHQ-2 and Brain Structure Stratified by Visual Acuity^a^

Outcome IDPs by quartile of visual acuity	Visual acuity, LogMAR	Coefficient (95% CI)	*P* value	*P* value for interaction
GMV in left supracalcarine cortex, mm^3^				
1	−0.48 to approximately −0.16	10.38 (1.03 to 15.67)	.006	.62
2	−0.16 to approximately −0.10	0.60 (−4.69 to 10.69)	.87	
3	−0.10 to approximately −0.02	7.16 (−0.02 to 14.30)	.051	
4	−0.02 to approximately 0.98	12.80 (4.50 to 19.85)	.001	
Mean ISOVF in the right fornix (cres) and/or stria terminalis, AU				
1	−0.48 to approximately −0.16	−3.5 × 10^−5^ (−2.8 × 10^−3^ to 2.7 × 10^−3^)	.98	.02
2	−0.16 to approximately −0.10	1.7 × 10^−3^ (−1.2 × 10^−3^ to 4.5 × 10^−3^)	.25	
3	−0.10 to approximately −0.02	4.6 × 10^−3^ (1.6 × 10^−3^ to 7.5 × 10^−3^)	.002	
4	−0.02 to approximately 0.98	5.8 × 10^−3^ (2.5 × 10^−3^ to 9.1 × 10^−3^)	.001	

Abbreviations: AU, arbitrary unit; GMV, gray matter volume; IDPs, imaging-derived phenotypes; ISOVF, isotropic volume fraction; LogMAR, logarithm of the minimum angle of resolution; PHQ, patient health questionnaire.

^a^ All models were adjusted for age, sex, race and ethnicity, Townsend index, educational qualifications, smoking, alcohol consumption, obesity, physical activity, history of hypertension, diabetes, hyperlipidemia, and family history of depression.

Sensitivity analyses defining depressive symptoms by PHQ-9 (n = 2194) showed the same pattern of association between PHQ-9 scores and mean ISOVF in the fornix (cres) and/or stria terminalis (eFigure 3A in the Supplement). We also observed an association between PHQ-9 scores and mean ISOVF in the fornix (cres) and/or stria terminalis in groups with poorer visual acuity (eFigure 3B in the Supplement). However, PHQ-9 score was not associated with GMV in the supracalcarine cortex after controlling for a range of covariates. The PHQ-9 score correlated with the PHQ-2 score assessed during the neuroimaging visit (Pearson *r* = 0.51; *P* < .001).

## Discussion

The findings of this large-scale cohort study suggest that poorer visual acuity was associated with depressive symptoms across the full spectrum. We also tested the association between depression and brain structures within different visual acuity subgroups and found a linear association between depressive symptoms and extracellular water diffusion in the fornix (cres) and/or stria terminalis in participants with poorer visual acuity. These findings suggest that visual health was associated with depressive symptoms and depression-related neurobiology.

The finding that poorer visual acuity is a potentially modifiable risk factor for depression is consistent with the epidemiological literature. Most previous studies were cross-sectional^13,35,36^ or focused on specific eye diseases.^37,38,39^ A longitudinal study from the Korean National Health Insurance Service^40^ showed that the risk of depression increased significantly in subgroups with visual impairment and blindness, with a hazard ratio of 1.31 in those with blindness. The Depression in Visual Impairment Trial^41,42^ suggested that problem-solving treatment and referring people to physicians were more effective than doing nothing for treating depression in patients with visual impairment. To conclude, previous evidence highlighted the need for interventions on the worst end of the visual spectrum (ie, visual impairment and blindness) rather than emphasizing the full continuum of visual acuity.

In the present study, 3.2% of the adults had impaired vision, and the prevalence of depression in visually impaired adults was 12.4%. The prevalence of depression was relatively lower in these data compared with current literature,^43,44,45^ tending to be influenced by variable subpopulations. To evaluate the full continuum of visual acuity, we considered it a continuous exposure in LogMAR units. We report an association of visual acuity with depression or only depressive symptoms measured by PHQ score. The evidence is independent in the axes of social differentiation with a trend across the full spectrum of visual acuity. Therefore, this finding suggests the potential of visual acuity correction in preventing underrecognized or preclinical depression and emphasizes the importance of regular visual health screening and liaising with mental health services to provide holistic care.

The present study considered IDPs generated from the T1-weighted structural MRI and dMRI, which have been used to study depression.^46,47,48^ To our knowledge, this is the first population-scale study to examine associations between depressive symptoms and brain IDPs while evaluating visual function. The association between PHQ-2 score and GMV in the supracalcarine cortex was detectable in the whole cohort. These findings align with those of a previous study^49^ that demonstrated the activation of calcarine area is correlated with stress tasks. Furthermore, the calcarine fissure has been hypothesized to be pathophysiological changes of depression.^50^ Last, the supracalcarine cortex is spatially connected to the primary visual cortex,^51^ suggesting the visual cortex may also be involved in the pathogenesis of depression. Although our results demonstrated that depressive symptoms were associated with volume of the supracalcarine cortex in the highest and lowest quartile of visual acuity, no significant modification was observed. Further studies are needed to demonstrate whether the supracalcarine cortex plays a vital role in the association between visual health and depressive symptoms.

Interestingly, we demonstrated dose-response–type gradients between PHQ score and ISOVF in the fornix (cres) and/or stria terminalis. Significantly higher ISOVF in the fornix (cres) and/or stria terminalis was suggestive of increased extracellular component of the free-water compartment. Studies on mental health supported our hypothesis that the fornix and stria terminalis are involved in the pathophysiology of schizophrenia, bipolar disorder, and autism spectrum disorder.^52,53^ Thus, our findings suggest that poorer visual acuity was associated with greater depressive symptoms and may have contributed to the related deterioration of the fornix and stria terminalis.

From a health policy perspective, finding ways to prevent depression and improve daily functioning has substantial public health impacts. Our findings highlight the value of visual health in association with mental health. Screening of vision at an early stage should be embedded in the middle-aged and older population to stratify the vulnerable population at risk for depression.

### Limitations

This study has some limitations. First, because there was no information on how long participants experienced visual impairment, we could not investigate whether results were affected by time. Second, depression may also affect vision.^54^ Further analysis should assess the causal relationships among visual health, depression, and brain changes. Third, the data set is limited by the large proportion of individuals who identified as White and therefore may not be generalizable to populations of racial and ethnic minority groups.

## Conclusions

The findings of this cohort study suggest that visual health was associated with depression in middle-aged and older individuals. The diffusion characteristic of ISOVF in the fornix (cres) and/or stria terminalis was associated with depressive symptoms in participants with poorer visual acuity.

## Notes

Supplement. **eMethods.** MRI Acquisition, IDPs Processing, and Demographic Data **eTable 1.** The 531 Brain MRI-Derived Phenotypes Derived by the UK Biobank Imaging Team **eTable 2.** Baseline Characteristics Stratified by Depression at Baseline **eTable 3.** Characteristics of Participants Included in the Neuroimaging Analysis **eTable 4.** Covariate-Adjusted Logistic Regression Analyses for the Associations Between Visual Function and Depression Stratified by Age and Sex **eTable 5.** Covariate-Adjusted Linear Regression Analyses to Evaluate Associations Between PHQ-2 Scores With Brain Macrostructures **eTable 6.** Covariate-Adjusted Linear Regression Analyses to Evaluate the Association Between PHQ-2 Scores With Brain Structure Stratified by Age and Sex **eFigure 1.** The Distribution of PHQ-2 Scores and Association With Visual Acuity **eFigure 2.** Linear Associations Between PHQ-2 Scores With Brain White Matter Microstructures **eFigure 3.** Associations Between PHQ-9 Scores and Mean ISOVF in the Right Fornix (cres) and/or Stria Terminalis
