# Supplementary material for: Association of Visual Health With Depressive Symptoms and Brain Imaging Phenotypes Among Middle-Aged and Older Adults
Source: JAMA Netw Open. 2022 Oct 6;5(10):e2235017. doi: 10.1001/jamanetworkopen.2022.35017 (PMC9539722; doi:10.1001/jamanetworkopen.2022.35017)
Supplement: Supplement. — eMethods. MRI Acquisition, IDPs Processing, and Demographic Data eTable 1. The 531 Brain MRI-Derived Phenotypes Derived by the UK Biobank Imaging Team eTable 2. Baseline Characteristics Stratified by Depression at Baseline eTable 3. Characteristics of Participants Included in the Neuroimaging Analysis eTable 4. Covariate-Adjusted Logistic Regression Analyses for the Associations Between Visual Function and Depression Stratified by Age and Sex eTable 5. Covariate-Adjusted Linear Regression Analyses to Evaluate Associations Between PHQ-2 Scores With Brain Macrostructures eTable 6. Covariate-Adjusted Linear Regression Analyses to Evaluate the Association Between PHQ-2 Scores With Brain Structure Stratified by Age and Sex eFigure 1. The Distribution of PHQ-2 Scores and Association With Visual Acuity eFigure 2. Linear Associations Between PHQ-2 Scores With Brain White Matter Microstructures eFigure 3. Associations Between PHQ-9 Scores and Mean ISOVF in the Right Fornix (cres) and/or Stria Terminalis [file jamanetwopen-e2235017-s001.pdf]

Supplementary Online Content

Zhang X, Shang X, Seth I, et al. Association of visual health with depressive symptoms and brain imaging phenotypes among middle-aged and older adults. *JAMA Netw Open*. 2022;5(10):e2235017. doi:10.1001/jamanetworkopen.2022.35017

eMethods. MRI Acquisition, IDPs Processing, and Demographic Data
eTable 1. The 531 Brain MRI-Derived Phenotypes Derived by the UK Biobank Imaging Team
eTable 2. Baseline Characteristics Stratified by Depression at Baseline
eTable 3. Characteristics of Participants Included in the Neuroimaging Analysis
eTable 4. Covariate-Adjusted Logistic Regression Analyses for the Associations Between Visual Function and Depression Stratified by Age and Sex
eTable 5. Covariate-Adjusted Linear Regression Analyses to Evaluate Associations Between PHQ-2 Scores With Brain Macrostructures
eTable 6. Covariate-Adjusted Linear Regression Analyses to Evaluate the Association Between PHQ-2 Scores With Brain Structure Stratified by Age and Sex
eFigure 1. The Distribution of PHQ-2 Scores and Association With Visual Acuity
eFigure 2. Linear Associations Between PHQ-2 Scores With Brain White Matter Microstructures
eFigure 3. Associations Between PHQ-9 Scores and Mean ISOVF in the Right Fornix (cres) and/or Stria Terminalis

This supplementary material has been provided by the authors to give readers additional information about their work.

© 2022 Zhang X et al. *JAMA Network Open*.

eMethods. MRI Acquisition, IDPs Processing, and Demographic Data

MRI acquisition and IDPs processing.

Brain MRI data were acquired commencing 4-6 years after baseline assessment on a standard Siemens Skyra 3T scanner (running VD13A SP4 software) with a Siemens 32-channel RF-receive head coil (eMethods). The imaging matrix was angled down by 16° from the AC-PC line. Total brain, white matter, grey matter, and regional grey matter volumes were acquired from T1 structural brain MRI. The microstructural of brain white matter showing cortical connection was quantified by dMRI. The dMRI protocol employed a spin-echo echo-planar imaging sequence with 10 T2-weighted ($b \approx 0$ s/mm^2) baseline, 50 $b = 1000$ s/mm^2 and 50 $b = 2000$ s/mm^2 diffusion-weighted volumes acquired with 100 distinct diffusion-encoding directions and three times multi-slice acquisition. The field of view was 104 × 104 mm, imaging matrix 52 × 52, 72 slices with a slice thickness of 2 mm, giving 2 mm isotropic voxels.

In addition to conventional measures of fractional anisotropy (FA; the directional coherence of water molecule diffusion) and mean diffusivity (MD; the magnitude of water molecule diffusion), the newer neurite orientation dispersion and density imaging (NODDI)[21] that measures greater information on the white matter microstructural was obtained.[22] NODDI provides estimates of neurite density (ICVF), extracellular water diffusion (ISOVF), and tract complexity/fanning (OD). We conducted analyses across all five water diffusion measures as discussed above (FA, MD, ICVF, ISOVF, and OD).

After the acquisition, images underwent an automated image processing pipeline developed by the UK Biobank, based on the FMRIB software library of MRI brain imaging data. The image acquisition and processing were conducted following the UK Biobank Brain Imaging Protocol (http://biobank.ctsu.ox.ac.uk/crystal/refer.cgi?id=2367), Brain Imaging Documentation (http://biobank.ctsu.ox.ac.uk/crystal/refer.cgi?id=1977). The resultant structural and water diffusion MRI parameters from these processing pipelines were derived by the UK Biobank Imaging team and made available as IDPs (eTable 1). There were 139 regional gray matter volume (GMV) IDPs derived using parcellations from the Harvard–Oxford cortical and subcortical atlases and Diedrichsen cerebellar atlas (UK Biobank field: 25782-25920). In addition, NODDI modeling of the dMRI data was conducted using the AMICO tool (Accelerated Microstructure Imaging via Convex Optimization; https://github.com/daducci/AMICO), a total of 375 white matter microstructure IDPs were derived (UK Biobank field: 25056-25151, 25344-25541, 25650-25730).

Participants with images badly affected by movement artifacts were removed by UK Biobank before the data release. In addition, 931 participants with a self-reported diagnosis of neurological diseases including stroke, dementia, Parkinson's disease, or any other demyelinating or neurodegenerative disorder were excluded. The flowchart in Figure 1 illustrates the inclusion of participants in the current study.

© 2022 Zhang X et al. *JAMA Network Open*.

Demographic data

Demographic information obtained included age, sex, and Townsend deprivation index (an area-based proxy measure for socioeconomic status). The ethnicity was self-reported (UK Biobank field: 21000) and recorded as white and non-white (Asian, Black, Chinese, Mixed, other ethnic group), with the genetic ancestry of 'White' also confirmed by genotypes (UK Biobank field: 22006).

Other covariates including educational qualifications, smoking, alcohol consumption, physical activity, and family history of severe depression were obtained through standardized questionnaire. Educational qualifications were obtained from standardized questionnaires using UK Biobank field: 6138. The data was recorded into a binary variable, indicating whether they held a university degree. Smoking status (UK Biobank field: 20116) and alcohol consumption (UK Biobank field: 20117) were recorded as current/previous or never. Physical activity levels (UK Biobank field: 22036) were recorded as above moderate/vigorous/walking or sedentary, according to International Physical Activity Questionnaire. Family history of depression (UK Biobank field: 20107, 20110, 20111, code 12) was recorded as first-degree relative diagnosis of severe depression or not.

Weight (kg) and height (m) were measured and used to calculate body mass index (BMI, weight (kg)/ height $(m)^2$). Obesity was defined as BMI (UK Biobank field: 21001) >30 kg/m^2. Diabetes mellitus was defined as self-reported (UK Biobank field: 20002), doctor-diagnosed diabetes mellitus (UK Biobank field: 2443), the use of anti-hyperglycemic medications or insulin (UK Biobank field: 20003, 6153), or with a glycated hemoglobin level ≥48 mmol/mol (UK Biobank field: 30750). Hypertension was defined as self-reported (UK Biobank field: 20002), the use of antihypertensive drugs (UK Biobank field: 6153), and average systolic blood pressure of at least 130mmHg or average diastolic blood pressure of at least 80mmHg (UK Biobank field: 4080). Hyperlipidemia was defined as self-reported (UK Biobank field: 20002), the use of hyperlipidemia-related medication or statins (UK Biobank field: 20003, 6153), or with a blood cholesterol level ≥ 6.21 mmol/L (UK Biobank field: 30690).

© 2022 Zhang X et al. *JAMA Network Open*.

eTable 1. The 531 Brain MRI-Derived Phenotypes Derived by the UK Biobank Imaging Team

UK Biobank Field ID	Description
25005	Volume of grey matter (normalised for head size)
25007	Volume of white matter (normalised for head size)
25009	Volume of brain, grey+white matter (normalised for head size)
25011	Volume of thalamus (left)
25012	Volume of thalamus (right)
25013	Volume of caudate (left)
25014	Volume of caudate (right)
25015	Volume of putamen (left)
25016	Volume of putamen (right)
25017	Volume of pallidum (left)
25018	Volume of pallidum (right)
25019	Volume of hippocampus (left)
25020	Volume of hippocampus (right)
25021	Volume of amygdala (left)
25022	Volume of amygdala (right)
25023	Volume of accumbens (left)
25024	Volume of accumbens (right)
25782	Volume of grey matter in Frontal Pole (left)
25783	Volume of grey matter in Frontal Pole (right)
25784	Volume of grey matter in Insular Cortex (left)
25785	Volume of grey matter in Insular Cortex (right)
25786	Volume of grey matter in Superior Frontal Gyrus (left)
25787	Volume of grey matter in Superior Frontal Gyrus (right)
25788	Volume of grey matter in Middle Frontal Gyrus (left)
25789	Volume of grey matter in Middle Frontal Gyrus (right)
25790	Volume of grey matter in Inferior Frontal Gyrus, pars triangularis (left)
25791	Volume of grey matter in Inferior Frontal Gyrus, pars triangularis (right)
25792	Volume of grey matter in Inferior Frontal Gyrus, pars opercularis (left)
25793	Volume of grey matter in Inferior Frontal Gyrus, pars opercularis (right)
25794	Volume of grey matter in Precentral Gyrus (left)
25795	Volume of grey matter in Precentral Gyrus (right)
25796	Volume of grey matter in Temporal Pole (left)
25797	Volume of grey matter in Temporal Pole (right)

© 2022 Zhang X et al. *JAMA Network Open*.

25798	Volume of grey matter in Superior Temporal Gyrus, anterior division (left)
25799	Volume of grey matter in Superior Temporal Gyrus, anterior division (right)
25800	Volume of grey matter in Superior Temporal Gyrus, posterior division (left)
25801	Volume of grey matter in Superior Temporal Gyrus, posterior division (right)
25802	Volume of grey matter in Middle Temporal Gyrus, anterior division (left)
25803	Volume of grey matter in Middle Temporal Gyrus, anterior division (right)
25804	Volume of grey matter in Middle Temporal Gyrus, posterior division (left)
25805	Volume of grey matter in Middle Temporal Gyrus, posterior division (right)
25806	Volume of grey matter in Middle Temporal Gyrus, temporooccipital part (left)
25807	Volume of grey matter in Middle Temporal Gyrus, temporooccipital part (right)
25808	Volume of grey matter in Inferior Temporal Gyrus, anterior division (left)
25809	Volume of grey matter in Inferior Temporal Gyrus, anterior division (right)
25810	Volume of grey matter in Inferior Temporal Gyrus, posterior division (left)
25811	Volume of grey matter in Inferior Temporal Gyrus, posterior division (right)
25812	Volume of grey matter in Inferior Temporal Gyrus, temporooccipital part (left)
25813	Volume of grey matter in Inferior Temporal Gyrus, temporooccipital part (right)
25814	Volume of grey matter in Postcentral Gyrus (left)
25815	Volume of grey matter in Postcentral Gyrus (right)
25816	Volume of grey matter in Superior Parietal Lobule (left)
25817	Volume of grey matter in Superior Parietal Lobule (right)
25818	Volume of grey matter in Supramarginal Gyrus, anterior division (left)
25819	Volume of grey matter in Supramarginal Gyrus, anterior division (right)
25820	Volume of grey matter in Supramarginal Gyrus, posterior division (left)
25821	Volume of grey matter in Supramarginal Gyrus, posterior division (right)
25822	Volume of grey matter in Angular Gyrus (left)
25823	Volume of grey matter in Angular Gyrus (right)
25824	Volume of grey matter in Lateral Occipital Cortex, superior division (left)
25825	Volume of grey matter in Lateral Occipital Cortex, superior division (right)
25826	Volume of grey matter in Lateral Occipital Cortex, inferior division (left)
25827	Volume of grey matter in Lateral Occipital Cortex, inferior division (right)
25828	Volume of grey matter in Intracalcarine Cortex (left)
25829	Volume of grey matter in Intracalcarine Cortex (right)
25830	Volume of grey matter in Frontal Medial Cortex (left)
25831	Volume of grey matter in Frontal Medial Cortex (right)
25832	Volume of grey matter in Juxtapositional Lobule Cortex (formerly Supplementary Motor Cortex) (left)

© 2022 Zhang X et al. *JAMA Network Open*.

25833	Volume of grey matter in Juxtapositional Lobule Cortex (formerly Supplementary Motor Cortex) (right)
25834	Volume of grey matter in Subcallosal Cortex (left)
25835	Volume of grey matter in Subcallosal Cortex (right)
25836	Volume of grey matter in Paracingulate Gyrus (left)
25837	Volume of grey matter in Paracingulate Gyrus (right)
25838	Volume of grey matter in Cingulate Gyrus, anterior division (left)
25839	Volume of grey matter in Cingulate Gyrus, anterior division (right)
25840	Volume of grey matter in Cingulate Gyrus, posterior division (left)
25841	Volume of grey matter in Cingulate Gyrus, posterior division (right)
25842	Volume of grey matter in Precuneous Cortex (left)
25843	Volume of grey matter in Precuneous Cortex (right)
25844	Volume of grey matter in Cuneal Cortex (left)
25845	Volume of grey matter in Cuneal Cortex (right)
25846	Volume of grey matter in Frontal Orbital Cortex (left)
25847	Volume of grey matter in Frontal Orbital Cortex (right)
25848	Volume of grey matter in Parahippocampal Gyrus, anterior division (left)
25849	Volume of grey matter in Parahippocampal Gyrus, anterior division (right)
25850	Volume of grey matter in Parahippocampal Gyrus, posterior division (left)
25851	Volume of grey matter in Parahippocampal Gyrus, posterior division (right)
25852	Volume of grey matter in Lingual Gyrus (left)
25853	Volume of grey matter in Lingual Gyrus (right)
25854	Volume of grey matter in Temporal Fusiform Cortex, anterior division (left)
25855	Volume of grey matter in Temporal Fusiform Cortex, anterior division (right)
25856	Volume of grey matter in Temporal Fusiform Cortex, posterior division (left)
25857	Volume of grey matter in Temporal Fusiform Cortex, posterior division (right)
25858	Volume of grey matter in Temporal Occipital Fusiform Cortex (left)
25859	Volume of grey matter in Temporal Occipital Fusiform Cortex (right)
25860	Volume of grey matter in Occipital Fusiform Gyrus (left)
25861	Volume of grey matter in Occipital Fusiform Gyrus (right)
25862	Volume of grey matter in Frontal Operculum Cortex (left)
25863	Volume of grey matter in Frontal Operculum Cortex (right)
25864	Volume of grey matter in Central Opercular Cortex (left)
25865	Volume of grey matter in Central Opercular Cortex (right)
25866	Volume of grey matter in Parietal Operculum Cortex (left)
25867	Volume of grey matter in Parietal Operculum Cortex (right)

© 2022 Zhang X et al. *JAMA Network Open*.

25868	Volume of grey matter in Planum Polare (left)
25869	Volume of grey matter in Planum Polare (right)
25870	Volume of grey matter in Heschl's Gyrus (includes H1 and H2) (left)
25871	Volume of grey matter in Heschl's Gyrus (includes H1 and H2) (right)
25872	Volume of grey matter in Planum Temporale (left)
25873	Volume of grey matter in Planum Temporale (right)
25874	Volume of grey matter in Supracalcarine Cortex (left)
25875	Volume of grey matter in Supracalcarine Cortex (right)
25876	Volume of grey matter in Occipital Pole (left)
25877	Volume of grey matter in Occipital Pole (right)
25878	Volume of grey matter in Thalamus (left)
25879	Volume of grey matter in Thalamus (right)
25880	Volume of grey matter in Caudate (left)
25881	Volume of grey matter in Caudate (right)
25882	Volume of grey matter in Putamen (left)
25883	Volume of grey matter in Putamen (right)
25884	Volume of grey matter in Pallidum (left)
25885	Volume of grey matter in Pallidum (right)
25886	Volume of grey matter in Hippocampus (left)
25887	Volume of grey matter in Hippocampus (right)
25888	Volume of grey matter in Amygdala (left)
25889	Volume of grey matter in Amygdala (right)
25890	Volume of grey matter in Ventral Striatum (left)
25891	Volume of grey matter in Ventral Striatum (right)
25892	Volume of grey matter in Brain-Stem
25893	Volume of grey matter in I-IV Cerebellum (left)
25894	Volume of grey matter in I-IV Cerebellum (right)
25895	Volume of grey matter in V Cerebellum (left)
25896	Volume of grey matter in V Cerebellum (right)
25897	Volume of grey matter in VI Cerebellum (left)
25898	Volume of grey matter in VI Cerebellum (vermis)
25899	Volume of grey matter in VI Cerebellum (right)
25900	Volume of grey matter in Crus I Cerebellum (left)
25901	Volume of grey matter in Crus I Cerebellum (vermis)
25902	Volume of grey matter in Crus I Cerebellum (right)
25903	Volume of grey matter in Crus II Cerebellum (left)

© 2022 Zhang X et al. *JAMA Network Open*.

25904	Volume of grey matter in Crus II Cerebellum (vermis)
25905	Volume of grey matter in Crus II Cerebellum (right)
25906	Volume of grey matter in VIIb Cerebellum (left)
25907	Volume of grey matter in VIIb Cerebellum (vermis)
25908	Volume of grey matter in VIIb Cerebellum (right)
25909	Volume of grey matter in VIIIa Cerebellum (left)
25910	Volume of grey matter in VIIIa Cerebellum (vermis)
25911	Volume of grey matter in VIIIa Cerebellum (right)
25912	Volume of grey matter in VIIIb Cerebellum (left)
25913	Volume of grey matter in VIIIb Cerebellum (vermis)
25914	Volume of grey matter in VIIIb Cerebellum (right)
25915	Volume of grey matter in IX Cerebellum (left)
25916	Volume of grey matter in IX Cerebellum (vermis)
25917	Volume of grey matter in IX Cerebellum (right)
25918	Volume of grey matter in X Cerebellum (left)
25919	Volume of grey matter in X Cerebellum (vermis)
25920	Volume of grey matter in X Cerebellum (right)
25056	Mean FA in middle cerebellar peduncle on FA skeleton
25057	Mean FA in pontine crossing tract on FA skeleton
25058	Mean FA in genu of corpus callosum on FA skeleton
25059	Mean FA in body of corpus callosum on FA skeleton
25060	Mean FA in splenium of corpus callosum on FA skeleton
25061	Mean FA in fornix on FA skeleton
25062	Mean FA in corticospinal tract on FA skeleton (right)
25063	Mean FA in corticospinal tract on FA skeleton (left)
25064	Mean FA in medial lemniscus on FA skeleton (right)
25065	Mean FA in medial lemniscus on FA skeleton (left)
25066	Mean FA in inferior cerebellar peduncle on FA skeleton (right)
25067	Mean FA in inferior cerebellar peduncle on FA skeleton (left)
25068	Mean FA in superior cerebellar peduncle on FA skeleton (right)
25069	Mean FA in superior cerebellar peduncle on FA skeleton (left)
25070	Mean FA in cerebral peduncle on FA skeleton (right)
25071	Mean FA in cerebral peduncle on FA skeleton (left)
25072	Mean FA in anterior limb of internal capsule on FA skeleton (right)
25073	Mean FA in anterior limb of internal capsule on FA skeleton (left)
25074	Mean FA in posterior limb of internal capsule on FA skeleton (right)

© 2022 Zhang X et al. *JAMA Network Open*.

25075	Mean FA in posterior limb of internal capsule on FA skeleton (left)
25076	Mean FA in retrolenticular part of internal capsule on FA skeleton (right)
25077	Mean FA in retrolenticular part of internal capsule on FA skeleton (left)
25078	Mean FA in anterior corona radiata on FA skeleton (right)
25079	Mean FA in anterior corona radiata on FA skeleton (left)
25080	Mean FA in superior corona radiata on FA skeleton (right)
25081	Mean FA in superior corona radiata on FA skeleton (left)
25082	Mean FA in posterior corona radiata on FA skeleton (right)
25083	Mean FA in posterior corona radiata on FA skeleton (left)
25084	Mean FA in posterior thalamic radiation on FA skeleton (right)
25085	Mean FA in posterior thalamic radiation on FA skeleton (left)
25086	Mean FA in sagittal stratum on FA skeleton (right)
25087	Mean FA in sagittal stratum on FA skeleton (left)
25088	Mean FA in external capsule on FA skeleton (right)
25089	Mean FA in external capsule on FA skeleton (left)
25090	Mean FA in cingulum cingulate gyrus on FA skeleton (right)
25091	Mean FA in cingulum cingulate gyrus on FA skeleton (left)
25092	Mean FA in cingulum hippocampus on FA skeleton (right)
25093	Mean FA in cingulum hippocampus on FA skeleton (left)
25094	Mean FA in fornix cres+stria terminalis on FA skeleton (right)
25095	Mean FA in fornix cres+stria terminalis on FA skeleton (left)
25096	Mean FA in superior longitudinal fasciculus on FA skeleton (right)
25097	Mean FA in superior longitudinal fasciculus on FA skeleton (left)
25098	Mean FA in superior fronto-occipital fasciculus on FA skeleton (right)
25099	Mean FA in superior fronto-occipital fasciculus on FA skeleton (left)
25100	Mean FA in uncinate fasciculus on FA skeleton (right)
25101	Mean FA in uncinate fasciculus on FA skeleton (left)
25102	Mean FA in tapetum on FA skeleton (right)
25103	Mean FA in tapetum on FA skeleton (left)
25104	Mean MD in middle cerebellar peduncle on FA skeleton
25105	Mean MD in pontine crossing tract on FA skeleton
25106	Mean MD in genu of corpus callosum on FA skeleton
25107	Mean MD in body of corpus callosum on FA skeleton
25108	Mean MD in splenium of corpus callosum on FA skeleton
25109	Mean MD in fornix on FA skeleton
25110	Mean MD in corticospinal tract on FA skeleton (right)

© 2022 Zhang X et al. *JAMA Network Open*.

25111	Mean MD in corticospinal tract on FA skeleton (left)
25112	Mean MD in medial lemniscus on FA skeleton (right)
25113	Mean MD in medial lemniscus on FA skeleton (left)
25114	Mean MD in inferior cerebellar peduncle on FA skeleton (right)
25115	Mean MD in inferior cerebellar peduncle on FA skeleton (left)
25116	Mean MD in superior cerebellar peduncle on FA skeleton (right)
25117	Mean MD in superior cerebellar peduncle on FA skeleton (left)
25118	Mean MD in cerebral peduncle on FA skeleton (right)
25119	Mean MD in cerebral peduncle on FA skeleton (left)
25120	Mean MD in anterior limb of internal capsule on FA skeleton (right)
25121	Mean MD in anterior limb of internal capsule on FA skeleton (left)
25122	Mean MD in posterior limb of internal capsule on FA skeleton (right)
25123	Mean MD in posterior limb of internal capsule on FA skeleton (left)
25124	Mean MD in retrolenticular part of internal capsule on FA skeleton (right)
25125	Mean MD in retrolenticular part of internal capsule on FA skeleton (left)
25126	Mean MD in anterior corona radiata on FA skeleton (right)
25127	Mean MD in anterior corona radiata on FA skeleton (left)
25128	Mean MD in superior corona radiata on FA skeleton (right)
25129	Mean MD in superior corona radiata on FA skeleton (left)
25130	Mean MD in posterior corona radiata on FA skeleton (right)
25131	Mean MD in posterior corona radiata on FA skeleton (left)
25132	Mean MD in posterior thalamic radiation on FA skeleton (right)
25133	Mean MD in posterior thalamic radiation on FA skeleton (left)
25134	Mean MD in sagittal stratum on FA skeleton (right)
25135	Mean MD in sagittal stratum on FA skeleton (left)
25136	Mean MD in external capsule on FA skeleton (right)
25137	Mean MD in external capsule on FA skeleton (left)
25138	Mean MD in cingulum cingulate gyrus on FA skeleton (right)
25139	Mean MD in cingulum cingulate gyrus on FA skeleton (left)
25140	Mean MD in cingulum hippocampus on FA skeleton (right)
25141	Mean MD in cingulum hippocampus on FA skeleton (left)
25142	Mean MD in fornix cres+stria terminalis on FA skeleton (right)
25143	Mean MD in fornix cres+stria terminalis on FA skeleton (left)
25144	Mean MD in superior longitudinal fasciculus on FA skeleton (right)
25145	Mean MD in superior longitudinal fasciculus on FA skeleton (left)
25146	Mean MD in superior fronto-occipital fasciculus on FA skeleton (right)

© 2022 Zhang X et al. *JAMA Network Open*.

25147	Mean MD in superior fronto-occipital fasciculus on FA skeleton (left)
25148	Mean MD in uncinate fasciculus on FA skeleton (right)
25149	Mean MD in uncinate fasciculus on FA skeleton (left)
25150	Mean MD in tapetum on FA skeleton (right)
25151	Mean MD in tapetum on FA skeleton (left)
25344	Mean ICVF in middle cerebellar peduncle on FA skeleton
25345	Mean ICVF in pontine crossing tract on FA skeleton
25346	Mean ICVF in genu of corpus callosum on FA skeleton
25347	Mean ICVF in body of corpus callosum on FA skeleton
25348	Mean ICVF in splenium of corpus callosum on FA skeleton
25349	Mean ICVF in fornix on FA skeleton
25350	Mean ICVF in corticospinal tract on FA skeleton (right)
25351	Mean ICVF in corticospinal tract on FA skeleton (left)
25352	Mean ICVF in medial lemniscus on FA skeleton (right)
25353	Mean ICVF in medial lemniscus on FA skeleton (left)
25354	Mean ICVF in inferior cerebellar peduncle on FA skeleton (right)
25355	Mean ICVF in inferior cerebellar peduncle on FA skeleton (left)
25356	Mean ICVF in superior cerebellar peduncle on FA skeleton (right)
25357	Mean ICVF in superior cerebellar peduncle on FA skeleton (left)
25358	Mean ICVF in cerebral peduncle on FA skeleton (right)
25359	Mean ICVF in cerebral peduncle on FA skeleton (left)
25360	Mean ICVF in anterior limb of internal capsule on FA skeleton (right)
25361	Mean ICVF in anterior limb of internal capsule on FA skeleton (left)
25362	Mean ICVF in posterior limb of internal capsule on FA skeleton (right)
25363	Mean ICVF in posterior limb of internal capsule on FA skeleton (left)
25364	Mean ICVF in retrolenticular part of internal capsule on FA skeleton (right)
25365	Mean ICVF in retrolenticular part of internal capsule on FA skeleton (left)
25366	Mean ICVF in anterior corona radiata on FA skeleton (right)
25367	Mean ICVF in anterior corona radiata on FA skeleton (left)
25368	Mean ICVF in superior corona radiata on FA skeleton (right)
25369	Mean ICVF in superior corona radiata on FA skeleton (left)
25370	Mean ICVF in posterior corona radiata on FA skeleton (right)
25371	Mean ICVF in posterior corona radiata on FA skeleton (left)
25372	Mean ICVF in posterior thalamic radiation on FA skeleton (right)
25373	Mean ICVF in posterior thalamic radiation on FA skeleton (left)
25374	Mean ICVF in sagittal stratum on FA skeleton (right)

© 2022 Zhang X et al. *JAMA Network Open*.

25375	Mean ICVF in sagittal stratum on FA skeleton (left)
25376	Mean ICVF in external capsule on FA skeleton (right)
25377	Mean ICVF in external capsule on FA skeleton (left)
25378	Mean ICVF in cingulum cingulate gyrus on FA skeleton (right)
25379	Mean ICVF in cingulum cingulate gyrus on FA skeleton (left)
25380	Mean ICVF in cingulum hippocampus on FA skeleton (right)
25381	Mean ICVF in cingulum hippocampus on FA skeleton (left)
25382	Mean ICVF in fornix cres+stria terminalis on FA skeleton (right)
25383	Mean ICVF in fornix cres+stria terminalis on FA skeleton (left)
25384	Mean ICVF in superior longitudinal fasciculus on FA skeleton (right)
25385	Mean ICVF in superior longitudinal fasciculus on FA skeleton (left)
25386	Mean ICVF in superior fronto-occipital fasciculus on FA skeleton (right)
25387	Mean ICVF in superior fronto-occipital fasciculus on FA skeleton (left)
25388	Mean ICVF in uncinate fasciculus on FA skeleton (right)
25389	Mean ICVF in uncinate fasciculus on FA skeleton (left)
25390	Mean ICVF in tapetum on FA skeleton (right)
25391	Mean ICVF in tapetum on FA skeleton (left)
25392	Mean OD in middle cerebellar peduncle on FA skeleton
25393	Mean OD in pontine crossing tract on FA skeleton
25394	Mean OD in genu of corpus callosum on FA skeleton
25395	Mean OD in body of corpus callosum on FA skeleton
25396	Mean OD in splenium of corpus callosum on FA skeleton
25397	Mean OD in fornix on FA skeleton
25398	Mean OD in corticospinal tract on FA skeleton (right)
25399	Mean OD in corticospinal tract on FA skeleton (left)
25400	Mean OD in medial lemniscus on FA skeleton (right)
25401	Mean OD in medial lemniscus on FA skeleton (left)
25402	Mean OD in inferior cerebellar peduncle on FA skeleton (right)
25403	Mean OD in inferior cerebellar peduncle on FA skeleton (left)
25404	Mean OD in superior cerebellar peduncle on FA skeleton (right)
25405	Mean OD in superior cerebellar peduncle on FA skeleton (left)
25406	Mean OD in cerebral peduncle on FA skeleton (right)
25407	Mean OD in cerebral peduncle on FA skeleton (left)
25408	Mean OD in anterior limb of internal capsule on FA skeleton (right)
25409	Mean OD in anterior limb of internal capsule on FA skeleton (left)
25410	Mean OD in posterior limb of internal capsule on FA skeleton (right)

© 2022 Zhang X et al. *JAMA Network Open*.

25411	Mean OD in posterior limb of internal capsule on FA skeleton (left)
25412	Mean OD in retrolenticular part of internal capsule on FA skeleton (right)
25413	Mean OD in retrolenticular part of internal capsule on FA skeleton (left)
25414	Mean OD in anterior corona radiata on FA skeleton (right)
25415	Mean OD in anterior corona radiata on FA skeleton (left)
25416	Mean OD in superior corona radiata on FA skeleton (right)
25417	Mean OD in superior corona radiata on FA skeleton (left)
25418	Mean OD in posterior corona radiata on FA skeleton (right)
25419	Mean OD in posterior corona radiata on FA skeleton (left)
25420	Mean OD in posterior thalamic radiation on FA skeleton (right)
25421	Mean OD in posterior thalamic radiation on FA skeleton (left)
25422	Mean OD in sagittal stratum on FA skeleton (right)
25423	Mean OD in sagittal stratum on FA skeleton (left)
25424	Mean OD in external capsule on FA skeleton (right)
25425	Mean OD in external capsule on FA skeleton (left)
25426	Mean OD in cingulum cingulate gyrus on FA skeleton (right)
25427	Mean OD in cingulum cingulate gyrus on FA skeleton (left)
25428	Mean OD in cingulum hippocampus on FA skeleton (right)
25429	Mean OD in cingulum hippocampus on FA skeleton (left)
25430	Mean OD in fornix cres+stria terminalis on FA skeleton (right)
25431	Mean OD in fornix cres+stria terminalis on FA skeleton (left)
25432	Mean OD in superior longitudinal fasciculus on FA skeleton (right)
25433	Mean OD in superior longitudinal fasciculus on FA skeleton (left)
25434	Mean OD in superior fronto-occipital fasciculus on FA skeleton (right)
25435	Mean OD in superior fronto-occipital fasciculus on FA skeleton (left)
25436	Mean OD in uncinate fasciculus on FA skeleton (right)
25437	Mean OD in uncinate fasciculus on FA skeleton (left)
25438	Mean OD in tapetum on FA skeleton (right)
25439	Mean OD in tapetum on FA skeleton (left)
25440	Mean ISOVF in middle cerebellar peduncle on FA skeleton
25441	Mean ISOVF in pontine crossing tract on FA skeleton
25442	Mean ISOVF in genu of corpus callosum on FA skeleton
25443	Mean ISOVF in body of corpus callosum on FA skeleton
25444	Mean ISOVF in splenium of corpus callosum on FA skeleton
25445	Mean ISOVF in fornix on FA skeleton
25446	Mean ISOVF in corticospinal tract on FA skeleton (right)

© 2022 Zhang X et al. *JAMA Network Open*.

25447	Mean ISOVF in corticospinal tract on FA skeleton (left)
25448	Mean ISOVF in medial lemniscus on FA skeleton (right)
25449	Mean ISOVF in medial lemniscus on FA skeleton (left)
25450	Mean ISOVF in inferior cerebellar peduncle on FA skeleton (right)
25451	Mean ISOVF in inferior cerebellar peduncle on FA skeleton (left)
25452	Mean ISOVF in superior cerebellar peduncle on FA skeleton (right)
25453	Mean ISOVF in superior cerebellar peduncle on FA skeleton (left)
25454	Mean ISOVF in cerebral peduncle on FA skeleton (right)
25455	Mean ISOVF in cerebral peduncle on FA skeleton (left)
25456	Mean ISOVF in anterior limb of internal capsule on FA skeleton (right)
25457	Mean ISOVF in anterior limb of internal capsule on FA skeleton (left)
25458	Mean ISOVF in posterior limb of internal capsule on FA skeleton (right)
25459	Mean ISOVF in posterior limb of internal capsule on FA skeleton (left)
25460	Mean ISOVF in retrolenticular part of internal capsule on FA skeleton (right)
25461	Mean ISOVF in retrolenticular part of internal capsule on FA skeleton (left)
25462	Mean ISOVF in anterior corona radiata on FA skeleton (right)
25463	Mean ISOVF in anterior corona radiata on FA skeleton (left)
25464	Mean ISOVF in superior corona radiata on FA skeleton (right)
25465	Mean ISOVF in superior corona radiata on FA skeleton (left)
25466	Mean ISOVF in posterior corona radiata on FA skeleton (right)
25467	Mean ISOVF in posterior corona radiata on FA skeleton (left)
25468	Mean ISOVF in posterior thalamic radiation on FA skeleton (right)
25469	Mean ISOVF in posterior thalamic radiation on FA skeleton (left)
25470	Mean ISOVF in sagittal stratum on FA skeleton (right)
25471	Mean ISOVF in sagittal stratum on FA skeleton (left)
25472	Mean ISOVF in external capsule on FA skeleton (right)
25473	Mean ISOVF in external capsule on FA skeleton (left)
25474	Mean ISOVF in cingulum cingulate gyrus on FA skeleton (right)
25475	Mean ISOVF in cingulum cingulate gyrus on FA skeleton (left)
25476	Mean ISOVF in cingulum hippocampus on FA skeleton (right)
25477	Mean ISOVF in cingulum hippocampus on FA skeleton (left)
25478	Mean ISOVF in fornix cres+stria terminalis on FA skeleton (right)
25479	Mean ISOVF in fornix cres+stria terminalis on FA skeleton (left)
25480	Mean ISOVF in superior longitudinal fasciculus on FA skeleton (right)
25481	Mean ISOVF in superior longitudinal fasciculus on FA skeleton (left)
25482	Mean ISOVF in superior fronto-occipital fasciculus on FA skeleton (right)

© 2022 Zhang X et al. *JAMA Network Open*.

25483	Mean ISOVF in superior fronto-occipital fasciculus on FA skeleton (left)
25484	Mean ISOVF in uncinate fasciculus on FA skeleton (right)
25485	Mean ISOVF in uncinate fasciculus on FA skeleton (left)
25486	Mean ISOVF in tapetum on FA skeleton (right)
25487	Mean ISOVF in tapetum on FA skeleton (left)
25488	Weighted-mean FA in tract acoustic radiation (left)
25489	Weighted-mean FA in tract acoustic radiation (right)
25490	Weighted-mean FA in tract anterior thalamic radiation (left)
25491	Weighted-mean FA in tract anterior thalamic radiation (right)
25492	Weighted-mean FA in tract cingulate gyrus part of cingulum (left)
25493	Weighted-mean FA in tract cingulate gyrus part of cingulum (right)
25494	Weighted-mean FA in tract parahippocampal part of cingulum (left)
25495	Weighted-mean FA in tract parahippocampal part of cingulum (right)
25496	Weighted-mean FA in tract corticospinal tract (left)
25497	Weighted-mean FA in tract corticospinal tract (right)
25498	Weighted-mean FA in tract forceps major
25499	Weighted-mean FA in tract forceps minor
25500	Weighted-mean FA in tract inferior fronto-occipital fasciculus (left)
25501	Weighted-mean FA in tract inferior fronto-occipital fasciculus (right)
25502	Weighted-mean FA in tract inferior longitudinal fasciculus (left)
25503	Weighted-mean FA in tract inferior longitudinal fasciculus (right)
25504	Weighted-mean FA in tract middle cerebellar peduncle
25505	Weighted-mean FA in tract medial lemniscus (left)
25506	Weighted-mean FA in tract medial lemniscus (right)
25507	Weighted-mean FA in tract posterior thalamic radiation (left)
25508	Weighted-mean FA in tract posterior thalamic radiation (right)
25509	Weighted-mean FA in tract superior longitudinal fasciculus (left)
25510	Weighted-mean FA in tract superior longitudinal fasciculus (right)
25511	Weighted-mean FA in tract superior thalamic radiation (left)
25512	Weighted-mean FA in tract superior thalamic radiation (right)
25513	Weighted-mean FA in tract uncinate fasciculus (left)
25514	Weighted-mean FA in tract uncinate fasciculus (right)
25515	Weighted-mean MD in tract acoustic radiation (left)
25516	Weighted-mean MD in tract acoustic radiation (right)
25517	Weighted-mean MD in tract anterior thalamic radiation (left)
25518	Weighted-mean MD in tract anterior thalamic radiation (right)

© 2022 Zhang X et al. *JAMA Network Open*.

25519	Weighted-mean MD in tract cingulate gyrus part of cingulum (left)
25520	Weighted-mean MD in tract cingulate gyrus part of cingulum (right)
25521	Weighted-mean MD in tract parahippocampal part of cingulum (left)
25522	Weighted-mean MD in tract parahippocampal part of cingulum (right)
25523	Weighted-mean MD in tract corticospinal tract (left)
25524	Weighted-mean MD in tract corticospinal tract (right)
25525	Weighted-mean MD in tract forceps major
25526	Weighted-mean MD in tract forceps minor
25527	Weighted-mean MD in tract inferior fronto-occipital fasciculus (left)
25528	Weighted-mean MD in tract inferior fronto-occipital fasciculus (right)
25529	Weighted-mean MD in tract inferior longitudinal fasciculus (left)
25530	Weighted-mean MD in tract inferior longitudinal fasciculus (right)
25531	Weighted-mean MD in tract middle cerebellar peduncle
25532	Weighted-mean MD in tract medial lemniscus (left)
25533	Weighted-mean MD in tract medial lemniscus (right)
25534	Weighted-mean MD in tract posterior thalamic radiation (left)
25535	Weighted-mean MD in tract posterior thalamic radiation (right)
25536	Weighted-mean MD in tract superior longitudinal fasciculus (left)
25537	Weighted-mean MD in tract superior longitudinal fasciculus (right)
25538	Weighted-mean MD in tract superior thalamic radiation (left)
25539	Weighted-mean MD in tract superior thalamic radiation (right)
25540	Weighted-mean MD in tract uncinate fasciculus (left)
25541	Weighted-mean MD in tract uncinate fasciculus (right)
25650	Weighted-mean ICVF in tract acoustic radiation (left)
25651	Weighted-mean ICVF in tract acoustic radiation (right)
25652	Weighted-mean ICVF in tract anterior thalamic radiation (left)
25653	Weighted-mean ICVF in tract anterior thalamic radiation (right)
25654	Weighted-mean ICVF in tract cingulate gyrus part of cingulum (left)
25655	Weighted-mean ICVF in tract cingulate gyrus part of cingulum (right)
25656	Weighted-mean ICVF in tract parahippocampal part of cingulum (left)
25657	Weighted-mean ICVF in tract parahippocampal part of cingulum (right)
25658	Weighted-mean ICVF in tract corticospinal tract (left)
25659	Weighted-mean ICVF in tract corticospinal tract (right)
25660	Weighted-mean ICVF in tract forceps major
25661	Weighted-mean ICVF in tract forceps minor
25662	Weighted-mean ICVF in tract inferior fronto-occipital fasciculus (left)

© 2022 Zhang X et al. *JAMA Network Open*.

25663	Weighted-mean ICVF in tract inferior fronto-occipital fasciculus (right)
25664	Weighted-mean ICVF in tract inferior longitudinal fasciculus (left)
25665	Weighted-mean ICVF in tract inferior longitudinal fasciculus (right)
25666	Weighted-mean ICVF in tract middle cerebellar peduncle
25667	Weighted-mean ICVF in tract medial lemniscus (left)
25668	Weighted-mean ICVF in tract medial lemniscus (right)
25669	Weighted-mean ICVF in tract posterior thalamic radiation (left)
25670	Weighted-mean ICVF in tract posterior thalamic radiation (right)
25671	Weighted-mean ICVF in tract superior longitudinal fasciculus (left)
25672	Weighted-mean ICVF in tract superior longitudinal fasciculus (right)
25673	Weighted-mean ICVF in tract superior thalamic radiation (left)
25674	Weighted-mean ICVF in tract superior thalamic radiation (right)
25675	Weighted-mean ICVF in tract uncinate fasciculus (left)
25676	Weighted-mean ICVF in tract uncinate fasciculus (right)
25677	Weighted-mean OD in tract acoustic radiation (left)
25678	Weighted-mean OD in tract acoustic radiation (right)
25679	Weighted-mean OD in tract anterior thalamic radiation (left)
25680	Weighted-mean OD in tract anterior thalamic radiation (right)
25681	Weighted-mean OD in tract cingulate gyrus part of cingulum (left)
25682	Weighted-mean OD in tract cingulate gyrus part of cingulum (right)
25683	Weighted-mean OD in tract parahippocampal part of cingulum (left)
25684	Weighted-mean OD in tract parahippocampal part of cingulum (right)
25685	Weighted-mean OD in tract corticospinal tract (left)
25686	Weighted-mean OD in tract corticospinal tract (right)
25687	Weighted-mean OD in tract forceps major
25688	Weighted-mean OD in tract forceps minor
25689	Weighted-mean OD in tract inferior fronto-occipital fasciculus (left)
25690	Weighted-mean OD in tract inferior fronto-occipital fasciculus (right)
25691	Weighted-mean OD in tract inferior longitudinal fasciculus (left)
25692	Weighted-mean OD in tract inferior longitudinal fasciculus (right)
25693	Weighted-mean OD in tract middle cerebellar peduncle
25694	Weighted-mean OD in tract medial lemniscus (left)
25695	Weighted-mean OD in tract medial lemniscus (right)
25696	Weighted-mean OD in tract posterior thalamic radiation (left)
25697	Weighted-mean OD in tract posterior thalamic radiation (right)
25698	Weighted-mean OD in tract superior longitudinal fasciculus (left)

© 2022 Zhang X et al. *JAMA Network Open*.

25699	Weighted-mean OD in tract superior longitudinal fasciculus (right)
25700	Weighted-mean OD in tract superior thalamic radiation (left)
25701	Weighted-mean OD in tract superior thalamic radiation (right)
25702	Weighted-mean OD in tract uncinate fasciculus (left)
25703	Weighted-mean OD in tract uncinate fasciculus (right)
25704	Weighted-mean ISOVF in tract acoustic radiation (left)
25705	Weighted-mean ISOVF in tract acoustic radiation (right)
25706	Weighted-mean ISOVF in tract anterior thalamic radiation (left)
25707	Weighted-mean ISOVF in tract anterior thalamic radiation (right)
25708	Weighted-mean ISOVF in tract cingulate gyrus part of cingulum (left)
25709	Weighted-mean ISOVF in tract cingulate gyrus part of cingulum (right)
25710	Weighted-mean ISOVF in tract parahippocampal part of cingulum (left)
25711	Weighted-mean ISOVF in tract parahippocampal part of cingulum (right)
25712	Weighted-mean ISOVF in tract corticospinal tract (left)
25713	Weighted-mean ISOVF in tract corticospinal tract (right)
25714	Weighted-mean ISOVF in tract forceps major
25715	Weighted-mean ISOVF in tract forceps minor
25716	Weighted-mean ISOVF in tract inferior fronto-occipital fasciculus (left)
25717	Weighted-mean ISOVF in tract inferior fronto-occipital fasciculus (right)
25718	Weighted-mean ISOVF in tract inferior longitudinal fasciculus (left)
25719	Weighted-mean ISOVF in tract inferior longitudinal fasciculus (right)
25720	Weighted-mean ISOVF in tract middle cerebellar peduncle
25721	Weighted-mean ISOVF in tract medial lemniscus (left)
25722	Weighted-mean ISOVF in tract medial lemniscus (right)
25723	Weighted-mean ISOVF in tract posterior thalamic radiation (left)
25724	Weighted-mean ISOVF in tract posterior thalamic radiation (right)
25725	Weighted-mean ISOVF in tract superior longitudinal fasciculus (left)
25726	Weighted-mean ISOVF in tract superior longitudinal fasciculus (right)
25727	Weighted-mean ISOVF in tract superior thalamic radiation (left)
25728	Weighted-mean ISOVF in tract superior thalamic radiation (right)
25729	Weighted-mean ISOVF in tract uncinate fasciculus (left)
25730	Weighted-mean ISOVF in tract uncinate fasciculus (right)

The image acquisition and processing were conducted in accordance with the UK Biobank Brain Imaging Protocol (http://biobank.ctsu.ox.ac.uk/crystal/refer.cgi?id=2367), Brain Imaging Documentation (http://biobank.ctsu.ox.ac.uk/crystal/refer.cgi?id=1977). We normalize all IDPs for head size by multiplying the raw IDP by the head size scaling factor.

© 2022 Zhang X et al. *JAMA Network Open*.

eTable 2. Baseline Characteristics Stratified by Depression at Baseline

Baseline Characteristics	Non-Depression Group	Depression Group [a]	OR (95% CI) [b]
N	103,083	11,500	-
Age, mean (SD), y	57.0 (8.1)	55.0 (8.0)	**0.97 (0.97-0.97)**
sex, No. (%)			
Female	55,309 (53.7)	7,092 (61.7)	1 [Reference]
Male	47,774 (46.4)	4,408 (38.3)	**0.73 (0.70-0.76)**
Ethnicity, No. (%) [c]			
White	92,810 (90.0)	9,500 (82.6)	1 [Reference]
Non-white	10,273 (10.0)	2,000 (17.4)	**1.72 (1.63-1.81)**
Townsend index, mean (SD)	-1.05 (2.95)	-0.76 (3.28)	**1.10 (1.09-1.11)**
Education level, No. (%)			
College or university degree	36,536 (35.4)	3,307 (28.8)	1 [Reference]
Others	66,547 (64.6)	8,193 (71.2)	**1.44 (1.38-1.50)**
Smoking status, No. (%)			
Never	57,394 (55.9)	5,938 (51.9)	1 [Reference]
Former/current	45,222 (44.1)	5,514 (48.1)	**1.28 (1.23-1.33)**
Drinking status, No. (%)			
Never	5,038 (4.9)	985 (8.6)	1 [Reference]
Former/current	97,855 (95.1)	10,482 (91.4)	**0.58 (0.54-0.63)**
Obesity, No. (%)			
No	78,324 (76.4)	7,694 (67.5)	1 [Reference]
Yes	24,213 (23.6)	3,708 (32.5)	**1.59 (1.53-1.66)**
Physical activity, No. (%)			
Not meeting recommendation	14,366 (17.0)	2,148 (24.0)	1 [Reference]
Meeting recommendation	69,997 (83.0)	6,793 (76.0)	**0.65 (0.62-0.69)**
Family history of depression, No. (%)			
No	90,611 (87.9)	8,689 (75.6)	1 [Reference]
Yes	12,472 (12.1)	2,811 (24.4)	**2.27 (2.17-2.38)**
History of diabetes, No. (%)			
No	96,652 (93.8)	10,374 (90.2)	1 [Reference]
Yes	6,431 (6.2)	1,126 (9.8)	**1.88 (1.76-2.01)**
History of hypertension, No. (%)			
No	26,274 (25.5)	3,142 (27.3)	1 [Reference]

© 2022 Zhang X et al. *JAMA Network Open*.

Yes	76,809 (74.5)	8,358 (72.7)	**1.10 (1.05-1.15)**
History of hyperlipidemia, No. (%)			
No	60,339 (58.5)	6,544 (56.9)	1 [Reference]
Yes	42,744 (41.5)	4,956 (43.1)	**1.18 (1.13-1.23)**

Bold values denote statistical significance at P <0.05 level. SD = standard deviation; OR = odds ratio; CI = confidence interval.

[a] Depression status was determined by a positive answer to the self-reported question, or a score of 3 or more on the PHQ-2 tool.

[b] Logistic regression models adjusted for age and sex.

[c] The ethnicity was self-reported and non-white includes Asian, Black, Chinese, Mixed, and other ethnic groups.

© 2022 Zhang X et al. *JAMA Network Open*.

eTable 3. Characteristics of Participants Included in the Neuroimaging Analysis

Variables	All	Females	Males
N	7,844	4,004	3,840
Age, mean (SD), y	55.5 (7.5)	55.0 (7.4)	56.1 (7.5)
PHQ-2 score, mean (SD)	0.39(0.89)	0.42(0.92)	0.36(0.86)
Visual acuity, mean (SD), logmar	-0.07 (0.13)	-0.07 (0.13)	-0.08 (0.13)
Total brain volume, mean (SD), cm^3	1497.2 (72.1)	1507.6 (72.0)	1486.4 (70.6)
Global GMV, mean (SD), cm^3	793.6 (47.0)	808.1 (45.2)	778.5 (44.0)
Global WMV, mean (SD), cm^3	703.6 (40.8)	699.5 (40.1)	707.9 (40.9)
Head size scaling factor: Mean (SD)	1.29 (0.12)	1.36 (0.10)	1.22 (0.09)

PHQ = patient health questionnaire; SD =standard deviation, GMV =gray matter volume, WMV = white matter volume.

© 2022 Zhang X et al. *JAMA Network Open*.

eTable 4. Covariate-Adjusted Logistic Regression Analyses for the Associations Between Visual Function and Depression Stratified by Age and Sex[a]

Age, y	Cases/ Population	Instrument	OR (95% CI) [b]	P Value	P for interaction
39-58	7,093/58,297	Visual acuity (Continuous variable, Per 0.1 LogMAR)	**1.05 (1.03-1.07)**	**<0.001**	0.55
		Visual impairment [c] (Categorical variable)	**1.28 (1.09-1.51)**	**0.001**	0.05
59-72	4,407/56,286	Visual acuity (Continuous variable, Per 0.1 LogMAR)	**1.06 (1.03-1.08)**	**<0.001**	1 [Reference]
		Visual impairment (Categorical variable)	1.07 (0.89-1.28)	0.336	1 [Reference]
**Sex**	**Cases/ Population**	**Instrument**	**OR (95% CI)**	**P Value**	**P for interaction**
Female	7,092/62,401	Visual acuity (Continuous variable, Per 0.1 LogMAR)	**1.06 (1.04-1.08)**	**<0.001**	0.86
		Visual impairment (Categorical variable)	**1.20 (1.03-1.40)**	**0.014**	0.85
Male	4,408/52,182	Visual acuity (Continuous variable, Per 0.1 LogMAR)	**1.05 (1.02-1.07)**	**<0.001**	1 [Reference]
		Visual impairment (Categorical variable)	1.17 (0.97-1.42)	0.092	1 [Reference]

[a] Depression was determined by a positive answer to the self-reported question, or a score of 3 or more on the PHQ-2 tool.

[b] Logistic regression was used to test the association between depression (Category variable) and visual impairment. All models have been adjusted for age, sex, ethnicity, Townsend index, educational qualifications, smoking, alcohol consumption, obesity, physical activity, history of hypertension, diabetes, hyperlipidemia, and family history of depression.

[c] No visual impairment defined as presenting visual acuity ≤ 0.3 LogMAR in better-seeing eye. Visual impairment defined as presenting visual acuity > 0.3 LogMAR in better-seeing eye.

Bold values denote statistical significance at p <0.05 level. SE = standard error; OR = odds ratio; CI = confidence interval; LogMAR = logarithm of the minimum angle of resolution.

© 2022 Zhang X et al. *JAMA Network Open*.

eTable 5. Covariate-Adjusted Linear Regression Analyses to Evaluate Associations Between PHQ-2 Scores With Brain Macrostructures

UK Biobank Field ID	Description	Multivariable Model 1			Multivariable Model 2		
		Coefficient	SE	Adjusted P Value	Coefficient	SE	Adjusted P Value
25005	Volume of grey matter (normalised for head size)	-373.52	455.10	0.83	-503.24	498.04	0.96
25007	Volume of white matter (normalised for head size)	765.47	497.35	0.41	621.76	549.74	0.93
25009	Volume of brain, grey+white matter (normalised for head size)	391.96	766.90	0.95	118.51	844.89	0.99
25011	Volume of thalamus (left)	-1.02	9.22	0.99	-2.85	10.14	0.99
25012	Volume of thalamus (right)	-4.50	8.77	0.95	-6.23	9.59	0.99
25013	Volume of caudate (left)	3.92	5.77	0.89	-1.03	6.38	0.99
25014	Volume of caudate (right)	0.43	6.11	0.99	-4.28	6.76	0.99
25015	Volume of putamen (left)	2.24	8.02	0.99	0.39	8.82	0.99
25016	Volume of putamen (right)	2.81	7.78	0.99	0.73	8.56	0.99
25017	Volume of pallidum (left)	-3.90	3.61	0.66	-4.76	4.00	0.93
25018	Volume of pallidum (right)	-3.54	3.64	0.74	-3.00	4.03	0.99
25019	Volume of hippocampus (left)	6.18	7.37	0.82	4.86	8.13	0.99
25020	Volume of hippocampus (right)	2.66	7.44	0.99	-0.06	8.15	1.00
25021	Volume of amygdala (left)	-7.20	3.85	0.32	-6.41	4.24	0.81
25022	Volume of amygdala (right)	1.88	4.27	0.99	0.11	4.69	0.99
25023	Volume of accumbens (left)	-2.12	1.76	0.59	-1.67	1.94	0.98

© 2022 Zhang X et al. *JAMA Network Open*.

25024	Volume of accumbens (right)	-1.02	1.62	0.91	-0.93	1.78	0.99
25782	Volume of grey matter in Frontal Pole (left)	-34.20	31.89	0.67	-50.51	35.19	0.83
25783	Volume of grey matter in Frontal Pole (right)	-47.40	34.73	0.50	-99.15	38.33	0.63
25784	Volume of grey matter in Insular Cortex (left)	-12.46	8.04	0.41	-19.70	8.84	0.63
25785	Volume of grey matter in Insular Cortex (right)	-2.35	8.13	0.99	-10.93	8.91	0.93
25786	Volume of grey matter in Superior Frontal Gyrus (left)	7.20	25.11	0.99	-3.40	27.53	0.99
25787	Volume of grey matter in Superior Frontal Gyrus (right)	-1.36	22.88	0.99	-5.89	25.11	0.99
25788	Volume of grey matter in Middle Frontal Gyrus (left)	1.47	25.63	0.99	-2.53	28.40	0.99
25789	Volume of grey matter in Middle Frontal Gyrus (right)	28.10	23.56	0.59	34.89	25.95	0.90
25790	Volume of grey matter in Inferior Frontal Gyrus, pars triangularis (left)	-11.84	9.15	0.54	-16.78	10.01	0.81
25791	Volume of grey matter in Inferior Frontal Gyrus, pars triangularis (right)	-3.88	7.91	0.96	-15.65	8.70	0.81
25792	Volume of grey matter in Inferior Frontal Gyrus, pars opercularis (left)	-9.50	8.23	0.62	-14.94	9.03	0.81
25793	Volume of grey matter in Inferior Frontal Gyrus, pars opercularis (right)	-9.38	8.25	0.63	-13.70	9.08	0.81
25794	Volume of grey matter in Precentral Gyrus (left)	13.19	24.27	0.95	6.22	26.59	0.99
25795	Volume of grey matter	-11.97	23.78	0.95	-6.23	26.12	0.99

© 2022 Zhang X et al. *JAMA Network Open*.

	in Precentral Gyrus (right)						
25796	Volume of grey matter in Temporal Pole (left)	-8.91	17.36	0.95	-21.15	19.10	0.93
25797	Volume of grey matter in Temporal Pole (right)	3.79	17.00	0.99	-14.45	18.69	0.99
25798	Volume of grey matter in Superior Temporal Gyrus, anterior division (left)	7.20	4.41	0.39	2.33	4.87	0.99
25799	Volume of grey matter in Superior Temporal Gyrus, anterior division (right)	-3.73	4.42	0.82	-6.38	4.88	0.91
25800	Volume of grey matter in Superior Temporal Gyrus, posterior division (left)	8.00	7.14	0.64	12.16	7.86	0.81
25801	Volume of grey matter in Superior Temporal Gyrus, posterior division (right)	-2.70	7.74	0.99	-4.41	8.56	0.99
25802	Volume of grey matter in Middle Temporal Gyrus, anterior division (left)	1.38	5.63	0.99	-5.18	6.20	0.98
25803	Volume of grey matter in Middle Temporal Gyrus, anterior division (right)	2.71	4.71	0.94	-0.72	5.21	0.99
25804	Volume of grey matter in Middle Temporal Gyrus, posterior division (left)	18.03	12.19	0.43	18.91	13.39	0.83
25805	Volume of grey matter in Middle Temporal Gyrus, posterior division (right)	-4.49	12.13	0.99	-9.21	13.31	0.99
25806	Volume of grey matter in Middle Temporal Gyrus, temporooccipital part	4.13	11.99	0.99	-8.57	13.25	0.99

© 2022 Zhang X et al. *JAMA Network Open*.

	(left)						
25807	Volume of grey matter in Middle Temporal Gyrus, temporooccipital part (right)	21.86	13.70	0.40	27.01	15.04	0.81
25808	Volume of grey matter in Inferior Temporal Gyrus, anterior division (left)	5.93	4.77	0.57	5.95	5.23	0.93
25809	Volume of grey matter in Inferior Temporal Gyrus, anterior division (right)	0.59	4.31	0.99	-0.42	4.74	0.99
25810	Volume of grey matter in Inferior Temporal Gyrus, posterior division (left)	1.29	11.23	0.99	-3.05	12.33	0.99
25811	Volume of grey matter in Inferior Temporal Gyrus, posterior division (right)	18.22	11.01	0.39	10.35	12.16	0.98
25812	Volume of grey matter in Inferior Temporal Gyrus, temporooccipital part (left)	-3.44	9.15	0.99	-9.59	10.09	0.98
25813	Volume of grey matter in Inferior Temporal Gyrus, temporooccipital part (right)	22.38	10.93	0.29	11.37	11.99	0.98
25814	Volume of grey matter in Postcentral Gyrus (left)	6.78	21.47	0.99	11.39	23.74	0.99
25815	Volume of grey matter in Postcentral Gyrus (right)	33.25	21.05	0.40	35.82	23.15	0.81
25816	Volume of grey matter in Superior Parietal Lobule (left)	-22.84	14.35	0.40	-20.05	15.87	0.92
25817	Volume of grey matter in Superior Parietal	-2.84	14.14	0.99	-7.19	15.61	0.99

© 2022 Zhang X et al. *JAMA Network Open*.

	Lobule (right)						
25818	Volume of grey matter in Supramarginal Gyrus, anterior division (left)	1.12	9.98	0.99	1.53	10.99	0.99
25819	Volume of grey matter in Supramarginal Gyrus, anterior division (right)	6.86	9.53	0.87	10.92	10.45	0.94
25820	Volume of grey matter in Supramarginal Gyrus, posterior division (left)	3.50	12.94	0.99	4.31	14.19	0.99
25821	Volume of grey matter in Supramarginal Gyrus, posterior division (right)	-11.55	15.77	0.87	-10.21	17.31	0.99
25822	Volume of grey matter in Angular Gyrus (left)	1.19	12.21	0.99	3.73	13.42	0.99
25823	Volume of grey matter in Angular Gyrus (right)	-15.19	16.92	0.78	-24.03	18.55	0.91
25824	Volume of grey matter in Lateral Occipital Cortex, superior division (left)	-28.78	30.92	0.77	-50.68	34.04	0.81
25825	Volume of grey matter in Lateral Occipital Cortex, superior division (right)	-10.98	31.33	0.99	-38.42	34.54	0.93
25826	Volume of grey matter in Lateral Occipital Cortex, inferior division (left)	29.83	17.57	0.38	12.51	19.31	0.99
25827	Volume of grey matter in Lateral Occipital Cortex, inferior division (right)	-10.59	18.44	0.94	-21.83	20.35	0.93
25828	Volume of grey matter in Intracalcarine Cortex (left)	12.30	9.31	0.52	14.99	10.28	0.82
25829	Volume of grey matter in Intracalcarine	13.59	8.77	0.41	17.63	9.69	0.81

© 2022 Zhang X et al. *JAMA Network Open*.

	Cortex (right)						
25830	Volume of grey matter in Frontal Medial Cortex (left)	-10.02	4.86	0.29	-10.67	5.37	0.81
25831	Volume of grey matter in Frontal Medial Cortex (right)	-4.20	5.00	0.82	-5.76	5.50	0.94
25832	Volume of grey matter in Juxtapositional Lobule Cortex (formerly Supplementary Motor Cortex) (left)	-16.58	8.55	0.30	-10.23	9.39	0.93
25833	Volume of grey matter in Juxtapositional Lobule Cortex (formerly Supplementary Motor Cortex) (right)	-18.89	8.55	0.27	-14.95	9.40	0.81
25834	Volume of grey matter in Subcallosal Cortex (left)	-9.25	4.96	0.32	-11.28	5.44	0.81
25835	Volume of grey matter in Subcallosal Cortex (right)	-6.84	4.41	0.41	-9.69	4.85	0.81
25836	Volume of grey matter in Paracingulate Gyrus (left)	8.77	11.29	0.85	-0.32	12.42	0.99
25837	Volume of grey matter in Paracingulate Gyrus (right)	3.94	11.60	0.99	-6.22	12.75	0.99
25838	Volume of grey matter in Cingulate Gyrus, anterior division (left)	-4.25	15.10	0.99	-19.62	16.70	0.93
25839	Volume of grey matter in Cingulate Gyrus, anterior division (right)	-6.51	16.70	0.99	-16.16	18.46	0.98
25840	Volume of grey matter in Cingulate Gyrus, posterior division (left)	-6.75	9.02	0.87	-9.30	9.84	0.98
25841	Volume of grey matter in Cingulate Gyrus, posterior division	-10.46	9.19	0.63	-14.96	10.03	0.81

© 2022 Zhang X et al. *JAMA Network Open*.

	(right)						
25842	Volume of grey matter in Precuneous Cortex (left)	6.74	17.46	0.99	3.37	19.18	0.99
25843	Volume of grey matter in Precuneous Cortex (right)	12.29	18.53	0.90	8.69	20.38	0.99
25844	Volume of grey matter in Cuneal Cortex (left)	10.93	5.90	0.32	8.87	6.52	0.89
25845	Volume of grey matter in Cuneal Cortex (right)	-0.95	6.80	0.99	-0.50	7.50	0.99
25846	Volume of grey matter in Frontal Orbital Cortex (left)	-5.82	10.77	0.95	-7.50	11.80	0.99
25847	Volume of grey matter in Frontal Orbital Cortex (right)	1.08	9.97	0.99	0.07	10.96	1.00
25848	Volume of grey matter in Parahippocampal Gyrus, anterior division (left)	0.37	5.97	0.99	-3.54	6.56	0.99
25849	Volume of grey matter in Parahippocampal Gyrus, anterior division (right)	-4.46	6.32	0.87	-8.11	6.96	0.93
25850	Volume of grey matter in Parahippocampal Gyrus, posterior division (left)	4.23	3.35	0.55	3.14	3.69	0.98
25851	Volume of grey matter in Parahippocampal Gyrus, posterior division (right)	0.16	2.78	0.99	-0.19	3.06	0.99
25852	Volume of grey matter in Lingual Gyrus (left)	2.94	11.15	0.99	-0.66	12.27	0.99
25853	Volume of grey matter in Lingual Gyrus (right)	-2.71	11.41	0.99	-2.73	12.49	0.99
25854	Volume of grey matter in Temporal Fusiform Cortex, anterior division (left)	-1.14	3.59	0.99	-1.26	3.96	0.99

© 2022 Zhang X et al. *JAMA Network Open*.

25855	Volume of grey matter in Temporal Fusiform Cortex, anterior division (right)	-4.42	3.22	0.50	-6.35	3.55	0.81
25856	Volume of grey matter in Temporal Fusiform Cortex, posterior division (left)	0.33	7.56	0.99	-3.41	8.32	0.99
25857	Volume of grey matter in Temporal Fusiform Cortex, posterior division (right)	1.19	6.48	0.99	-3.13	7.15	0.99
25858	Volume of grey matter in Temporal Occipital Fusiform Cortex (left)	4.24	6.76	0.91	4.07	7.46	0.99
25859	Volume of grey matter in Temporal Occipital Fusiform Cortex (right)	5.74	8.42	0.89	7.52	9.25	0.98
25860	Volume of grey matter in Occipital Fusiform Gyrus (left)	2.83	8.92	0.99	4.35	9.83	0.99
25861	Volume of grey matter in Occipital Fusiform Gyrus (right)	1.58	8.76	0.99	-0.89	9.68	0.99
25862	Volume of grey matter in Frontal Operculum Cortex (left)	-8.13	3.80	0.29	-10.55	4.16	0.63
25863	Volume of grey matter in Frontal Operculum Cortex (right)	-1.55	3.63	0.99	-5.57	3.98	0.84
25864	Volume of grey matter in Central Opercular Cortex (left)	-2.35	7.58	0.99	-7.47	8.32	0.98
25865	Volume of grey matter in Central Opercular Cortex (right)	-8.32	7.88	0.68	-9.01	8.66	0.94
25866	Volume of grey matter in Parietal Operculum Cortex (left)	1.59	6.43	0.99	1.83	7.06	0.99
25867	Volume of grey matter in Parietal Operculum Cortex (right)	-4.72	5.98	0.84	-2.56	6.59	0.99

© 2022 Zhang X et al. *JAMA Network Open*.

25868	Volume of grey matter in Planum Polare (left)	0.24	2.97	0.99	-2.50	3.27	0.99
25869	Volume of grey matter in Planum Polare (right)	-3.62	3.00	0.59	-3.11	3.30	0.98
25870	Volume of grey matter in Heschl's Gyrus (includes H1 and H2) (left)	-0.94	3.75	0.99	-0.37	4.14	0.99
25871	Volume of grey matter in Heschl's Gyrus (includes H1 and H2) (right)	-0.01	2.77	1.00	0.66	3.05	0.99
25872	Volume of grey matter in Planum Temporale (left)	5.18	6.71	0.85	7.94	7.40	0.93
25873	Volume of grey matter in Planum Temporale (right)	0.54	4.37	0.99	0.14	4.82	0.99
**25874**	**Volume of grey matter in Supracalcarine Cortex (left)**	**6.15**	**1.72**	**0.01**	**7.61**	**1.89**	**0.01**
25875	Volume of grey matter in Supracalcarine Cortex (right)	3.00	2.37	0.55	4.07	2.62	0.81
25876	Volume of grey matter in Occipital Pole (left)	-6.51	20.13	0.99	3.65	22.12	0.99
25877	Volume of grey matter in Occipital Pole (right)	31.57	19.05	0.39	39.64	20.92	0.81
25878	Volume of grey matter in Thalamus (left)	2.38	4.46	0.95	3.36	4.86	0.99
25879	Volume of grey matter in Thalamus (right)	2.97	4.61	0.91	3.19	5.04	0.99
25880	Volume of grey matter in Caudate (left)	30.73	10.21	0.19	25.83	11.31	0.63
25881	Volume of grey matter in Caudate (right)	29.79	10.56	0.19	27.67	11.63	0.63
25882	Volume of grey matter in Putamen (left)	11.47	6.75	0.38	9.26	7.41	0.93
25883	Volume of grey matter in Putamen (right)	12.35	7.29	0.38	10.59	7.99	0.91

© 2022 Zhang X et al. *JAMA Network Open*.

25884	Volume of grey matter in Pallidum (left)	-0.24	0.62	0.99	-0.12	0.69	0.99
25885	Volume of grey matter in Pallidum (right)	-0.42	0.68	0.92	-0.12	0.75	0.99
25886	Volume of grey matter in Hippocampus (left)	5.13	5.77	0.79	4.77	6.36	0.99
25887	Volume of grey matter in Hippocampus (right)	0.77	6.03	0.99	-1.69	6.61	0.99
25888	Volume of grey matter in Amygdala (left)	-4.21	3.16	0.51	-5.48	3.49	0.81
25889	Volume of grey matter in Amygdala (right)	-6.97	3.46	0.29	-9.37	3.80	0.63
25890	Volume of grey matter in Ventral Striatum (left)	-0.03	1.60	0.99	-0.16	1.76	0.99
25891	Volume of grey matter in Ventral Striatum (right)	0.05	1.59	0.99	0.11	1.75	0.99
25892	Volume of grey matter in Brain-Stem	8.36	12.13	0.89	15.44	12.72	0.93
25893	Volume of grey matter in I-IV Cerebellum (left)	-8.30	4.37	0.32	-7.61	4.78	0.81
25894	Volume of grey matter in I-IV Cerebellum (right)	-9.97	4.75	0.29	-10.40	5.21	0.81
25895	Volume of grey matter in V Cerebellum (left)	-12.44	5.69	0.28	-10.57	6.27	0.81
25896	Volume of grey matter in V Cerebellum (right)	-9.99	5.54	0.34	-10.13	6.07	0.81
25897	Volume of grey matter in VI Cerebellum (left)	-33.64	14.90	0.26	-27.69	16.38	0.81
25898	Volume of grey matter in VI Cerebellum (vermis)	-3.53	3.80	0.77	-2.89	4.18	0.99
25899	Volume of grey matter in VI Cerebellum (right)	-20.18	14.55	0.49	-11.52	15.93	0.99
25900	Volume of grey matter in Crus I Cerebellum (left)	-49.44	24.27	0.29	-33.41	26.63	0.93
25901	Volume of grey matter in Crus I Cerebellum	-0.01	0.03	0.99	-0.01	0.03	0.99

© 2022 Zhang X et al. *JAMA Network Open*.

	(vermis)						
25902	Volume of grey matter in Crus I Cerebellum (right)	-55.68	25.65	0.28	-45.52	28.15	0.81
25903	Volume of grey matter in Crus II Cerebellum (left)	-24.00	18.18	0.52	-18.44	19.93	0.98
25904	Volume of grey matter in Crus II Cerebellum (vermis)	-1.95	1.28	0.41	-2.24	1.41	0.81
25905	Volume of grey matter in Crus II Cerebellum (right)	-24.62	17.54	0.48	-13.22	19.24	0.99
25906	Volume of grey matter in VIIb Cerebellum (left)	-19.11	9.96	0.31	-15.64	10.93	0.83
25907	Volume of grey matter in VIIb Cerebellum (vermis)	-1.16	0.45	0.20	-1.07	0.49	0.65
25908	Volume of grey matter in VIIb Cerebellum (right)	-16.71	10.51	0.40	-7.39	11.58	0.99
25909	Volume of grey matter in VIIIa Cerebellum (left)	-27.61	10.24	0.20	-25.30	11.31	0.63
25910	Volume of grey matter in VIIIa Cerebellum (vermis)	-4.57	2.64	0.37	-4.72	2.91	0.81
25911	Volume of grey matter in VIIIa Cerebellum (right)	-29.92	10.87	0.19	-24.12	11.96	0.81
25912	Volume of grey matter in VIIIb Cerebellum (left)	-15.51	7.69	0.29	-12.69	8.45	0.81
25913	Volume of grey matter in VIIIb Cerebellum (vermis)	-1.65	1.35	0.57	-1.14	1.48	0.99
25914	Volume of grey matter in VIIIb Cerebellum (right)	-16.69	8.35	0.29	-14.87	9.15	0.81
25915	Volume of grey matter in IX Cerebellum (left)	-9.68	6.10	0.40	-5.72	6.68	0.98
25916	Volume of grey matter	-1.40	1.41	0.73	-1.28	1.56	0.98

© 2022 Zhang X et al. *JAMA Network Open*.

	in IX Cerebellum (vermis)						
25917	Volume of grey matter in IX Cerebellum (right)	-10.41	6.69	0.41	-6.25	7.35	0.98
25918	Volume of grey matter in X Cerebellum (left)	0.01	1.23	1.00	0.70	1.36	0.99
25919	Volume of grey matter in X Cerebellum (vermis)	-0.87	0.70	0.57	-0.87	0.77	0.93
25920	Volume of grey matter in X Cerebellum (right)	0.80	1.22	0.91	0.92	1.34	0.99

Model 1 has been adjusted for age, sex, ethnicity, and obesity; model 2 has been adjusted for age, sex, ethnicity, obesity, Townsend index, educational qualifications, smoking, alcohol consumption, physical activity, history of hypertension, diabetes, hyperlipidemia, and family history of depression. Adjusted P-values were computed using False Discovery Rate methods. Bold values denote statistical significance at P adjusted < 0.05 level. SE = standard error; PHQ = patient health questionnaire.

© 2022 Zhang X et al. *JAMA Network Open*.

eTable 6. Covariate-Adjusted Linear Regression Analyses to Evaluate the Association Between PHQ-2 Scores With Brain Structure Stratified by Age and Sex

Age, y [a]	Cases/ Population	Outcome	Coefficient (95% CI) [b]	P Value	P for interaction
44-64	4,155/7,844	Volume of grey matter in the left supracalcarine cortex	**7.46 (2.98-11.93)**	**0.001**	0.58
		Mean ISOVF in the right fornix (cres)/stria terminalis	**0.003 (0.001-0.004)**	**0.001**	0.47
64-79	3,689/7,844	Volume of grey matter in the left supracalcarine cortex	**7.08 (0.44-1.51)**	**0.037**	1 [Reference]
		Mean ISOVF in the right fornix (cres)/stria terminalis	0.003 (-0.0002-0.006)	0.076	1 [Reference]
**Sex**	**Cases/ Population**	**Outcome**	**Coefficient (95% CI)**	**P Value**	**P for interaction**
Female	4,004/7,844	Volume of grey matter in the left supracalcarine cortex	**10.04 (5.00-15.09)**	**<0.001**	**0.030**
		Mean ISOVF in the right fornix (cres)/stria terminalis	0.001 (-0.001-0.003)	0.244	**0.041**
Male	3,840/7,844	Volume of grey matter in the left supracalcarine cortex	4.30 (-1.16-9.76)	0.123	1 [Reference]
		Mean ISOVF in the right fornix (cres)/stria terminalis	**0.005 (0.002-0.007)**	**<0.001**	1 [Reference]

[a] Age (median = 64 years) was obtained by the time when participants attended assessment centre at the imaging visit after 2014.

[b] Linear regression models were used to test associations between PHQ-2 scores with brain structures. All models have been adjusted for age, sex, ethnicity, Townsend index, educational qualifications, smoking, alcohol consumption, obesity, physical activity, history of hypertension, diabetes, hyperlipidemia, and family history of depression.

Bold values denote statistical significance at p <0.05 level. CI = confidence interval; ISOVF, isotropic volume fraction.

© 2022 Zhang X et al. *JAMA Network Open*.

eFigure 1. The Distribution of PHQ-2 Scores and Association With Visual Acuity

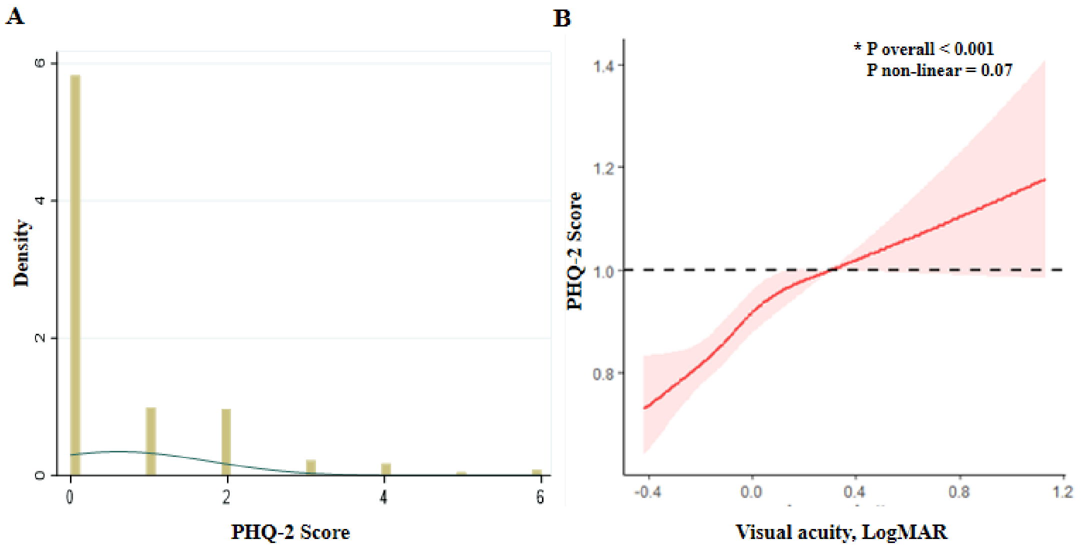

(A)The distribution of PHQ-2 scores is positively skewed. (B) The model was fitted with a restricted cubic spline for visual acuity, adjusted for age, sex, ethnicity, Townsend index, educational qualifications, smoking, alcohol consumption, obesity, physical activity, history of hypertension, diabetes, hyperlipidemia, and family history of depression. The reference visual acuity was 0.3 LogMAR. Evidence of overll association between visual acuity and PHQ-2 score was observed instead of any nonlinear association (P overall < 0.001; P non-linear > 0.05). *P < 0.05 level.

© 2022 Zhang X et al. *JAMA Network Open*.

eFigure 2. Linear Associations Between PHQ-2 Scores With Brain White Matter Microstructures

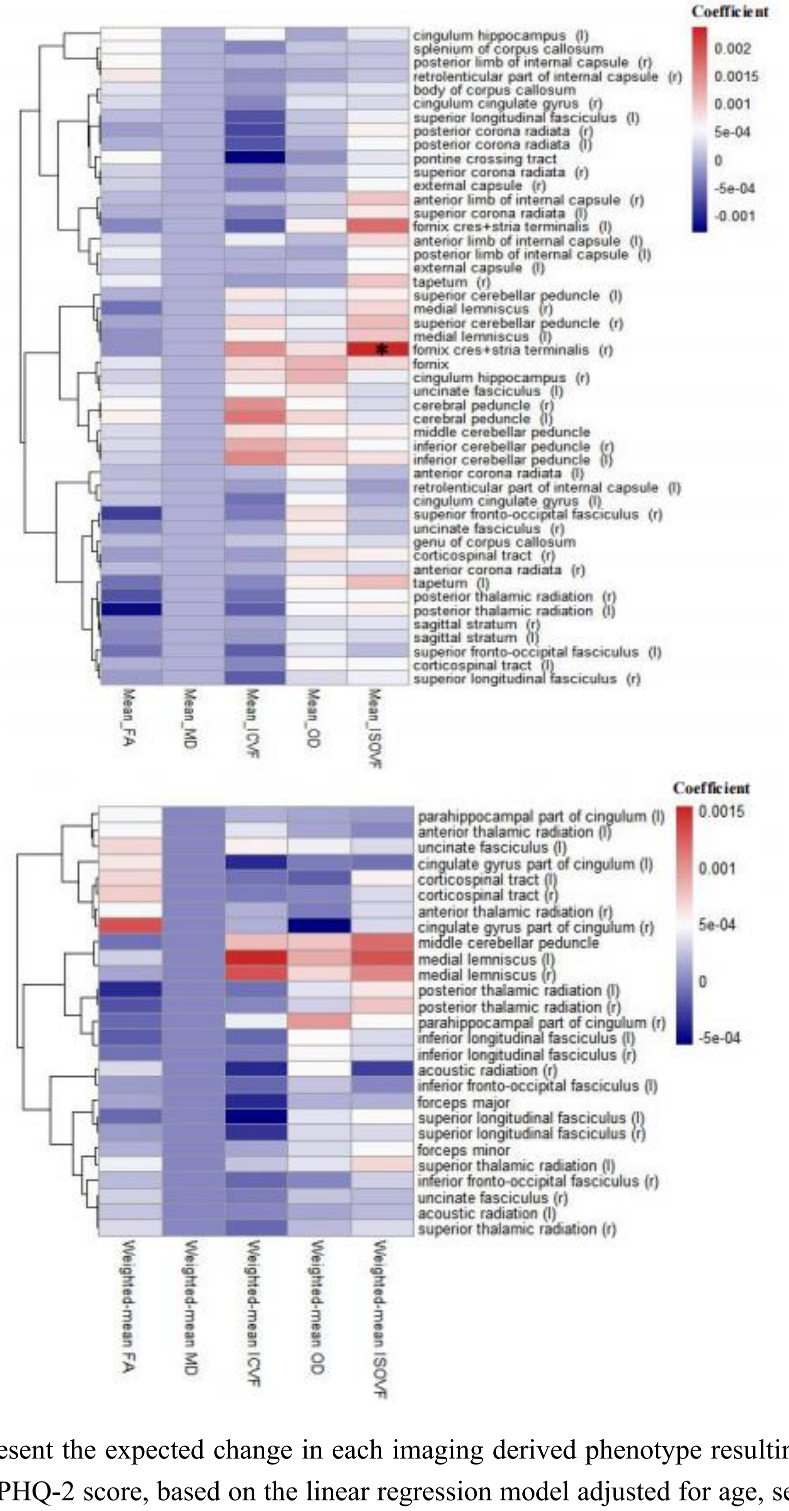

Colors represent the expected change in each imaging derived phenotype resulting from the each increase in PHQ-2 score, based on the linear regression model adjusted for age, sex, ethnicity, and obesity. FA = fractional anisotropy, ICVF = intracellular volume fraction, ISOVF = isotropic volume fraction, MD = mean diffusivity, OD = orientation dispersion, r = right, l = left. * Adjusted $P < 0.05$ level.

© 2022 Zhang X et al. *JAMA Network Open*.

eFigure 3. Associations Between PHQ-9 Scores and Mean ISOVF in the Right Fornix (cres) and/or Stria Terminalis

A

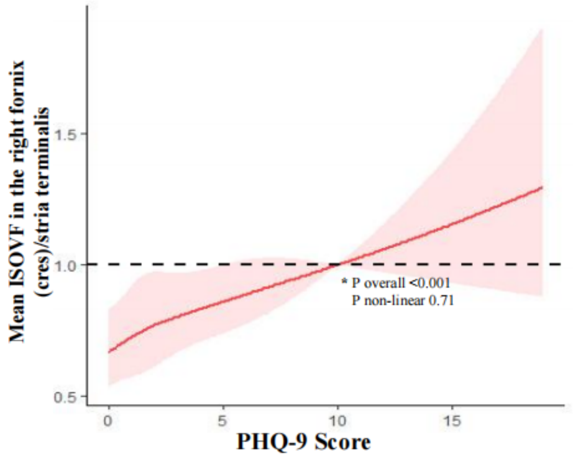

B

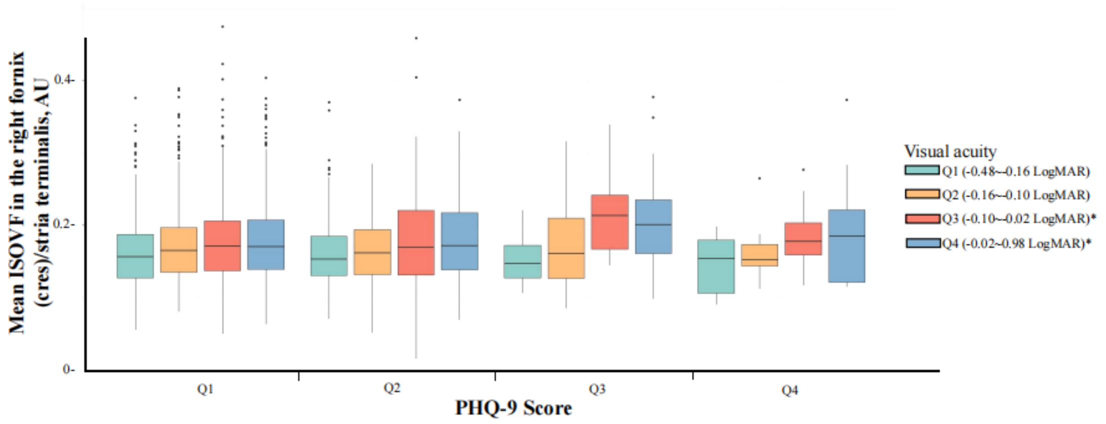

(A) The models were fitted with a restricted cubic spline for PHQ-9 score, adjusted for age, sex, ethnicity, Townsend index, educational qualifications, smoking, alcohol consumption, obesity, physical activity, history of hypertension, diabetes, hyperlipidemia, and family history of depression. The reference PHQ-9 score was 10. Evidence of positive associations between PHQ-9 score and greater ISOVF in the right fornix (cres)/stria terminalis was observed (P overall < 0.001; P non-linear > 0.1). (B) Association between PHQ-9 score and mean ISOVF in the right fornix (cres)/stria terminalis, stratified by visual function. Among those with the higher visual loss (highest 2 quartiles), greater depression score was associated with higher ISOVF levels. OR = odds ratio; CI = confidence interval; LogMAR = logarithm of the minimum angle of resolution; PHQ = patient health questionnaire. * P < 0.05 level.

© 2022 Zhang X et al. *JAMA Network Open*.
